# Scutellarin attenuated tubule cell apoptosis by modulating HIF-1α for the treatment of DKD: the insight integrating network analysis, machine learning and single-cell transcriptome

**DOI:** 10.3389/fphar.2025.1656409

**Published:** 2025-09-30

**Authors:** Li Jiang, Jie Jian, Haojun Zhang, Xiai Wu

**Affiliations:** ^1^ Diabetes Department of Integrated Chinese and Western Medicine, China National Center for Integrated Traditional Chinese and Western Medicine, China-Japan Friendship Hospital, Beijing, China; ^2^ Department of Internal Medicine, Mental Health Center of Dongcheng District, Beijing, China; ^3^ Beijing Key Lab for Immune-Mediated Inflammatory Diseases, Institute of Clinical Medical Sciences, China-Japan Friendship Hospital, Beijing, China

**Keywords:** diabetic kidney disease, scutellarin, machine learning, single-cell transcriptome analysis, network analysis

## Abstract

**Aim:**

To explore the possible mechanism and target of scutellarin (Scu) on diabetic kidney disease (DKD).

**Method:**

The Network analysis was used to explore and enrich the possible pathway. RNA transcriptome were employed to deepen the understanding of candidate targets in key signaling pathways. Core targets were optimized through 8 machine learning algorithms. Single-cell transcriptome were utilized to clarify the expression locations and temporal trajectories of core targets, identifying high-expression cell types. Finally, molecular docking and cell experiments were conducted to validate the regulatory effects of Scutellarin on the molecular targets.

**Result:**

The Network analysis showed the roles of hypoxic response and apoptosis pathways. RNA transcriptome and machine learning identified HIF-1α and CASP3 as the hub genes related to DKD outcomes and hypoxic apoptosis. Single-cell transcriptome analysis confirmed the expression patterns and locations of hub genes, identifying the CD-PC cells as the high-expression cell type. *In-vitro* experiments demonstrated 20 μM scutellarin was most beneficial for mIMCD-3 cell proliferation. The hypoxia significantly enhanced HIF-1α gene transcription driven by HRE conserved genes (P < 0.0001), whereas high glucose inhibited hypoxia-induced HIF-1α transcription (P < 0.05). Scutellarin significantly upregulated HIF-1α transcriptional activity (P < 0.05) and HIF-1α total protein expression under high glucose-hypoxia (P < 0.05), reduced mitochondrial ROS release (P < 0.05) and renal tubular cell apoptosis (P < 0.01).

**Conclusion:**

Scutellarin attenuated renal collecting duct cell apoptosis by modulating HIF-1α for the treatment of DKD.

## Graphical Abstract

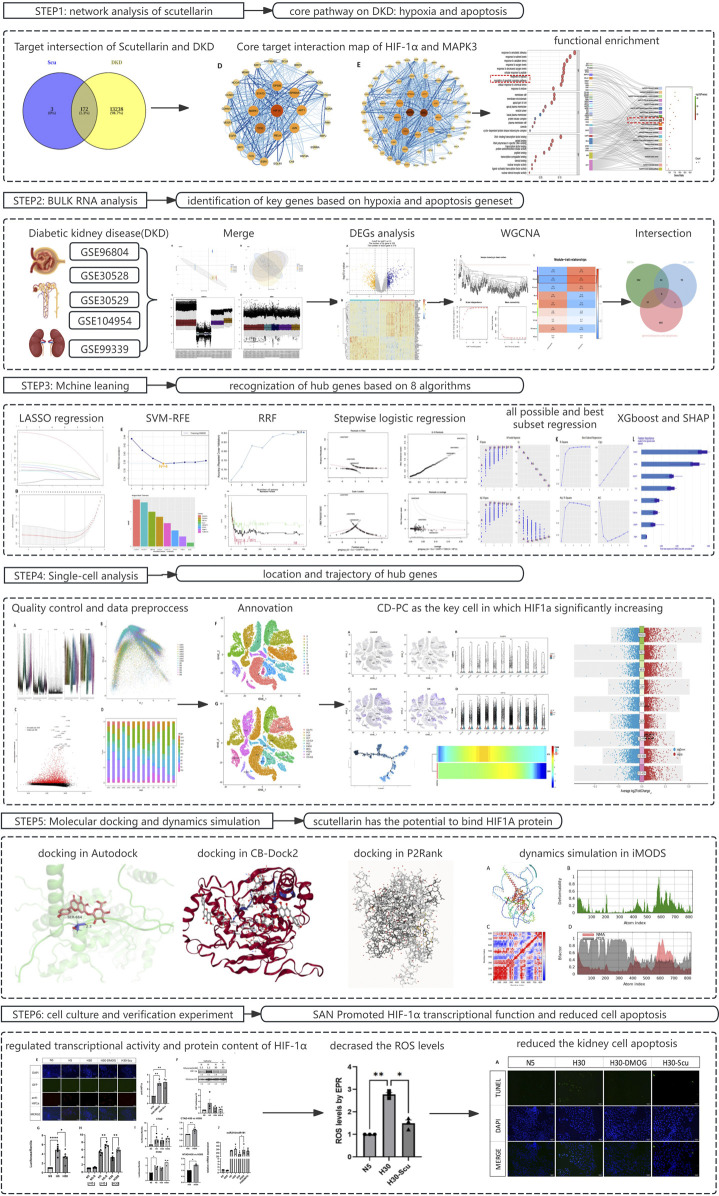

## 1 Introduction

Diabetic Kidney Disease (DKD) is a renal disease secondary to diabetes mellitus, which is mainly manifested by an increased urinary albumin excretion rate with a decreased glomerular filtration rate (Thomas et al., 2015). It affected approximately 35%–40% of all individuals with diabetes and accounting for roughly half of incident dialysis cases in high-income countries (Liu et al., 2024). Despite the implementation of renin-angiotensin system blockade, SGLT-2 inhibitors, mineralocorticoid receptor antagonists, and stringent glycaemic control, 15%–25% of patients still progress to ESRD, highlighting an urgent need for additional mechanism-based therapies (Barutta et al., 2021; Chen et al., 2020).

Traditional Chinese Medicine (TCM) has garnered significant attention in the treatment of DKD due to its holistic approach, focusing on anti-inflammatory effects, regulation of DNA methylation and improvement of the intestinal micro-environment (Jiang et al., 2022; Ma et al., 2024; Zhao et al., 2024). *Erigeron breviscapus* was the commonly used herb which have been shown the certain hypoglycaemic and nephroprotective effects (Wu et al., 2021; Qi et al., 2006; Xu et al., 2013). Scutellarin (4′,5,6,7-tetrahydroxyflavone-7-O-glucuronide), a major bioactive constituent and the quality control of *E. breviscapus* herbs (National Pharmacopoeia Committee, 2020), has been verified to have a wide range of well-established pharmacological activities, including improving microcirculation, antioxidant, antifibrotic, and others (Wang and Ma, 2018). More essentially, it has been proven to ameliorate various features of DKD *in vivo*, including proteinuria, glomerular expansion, mesangial matrix accumulation, renal fibrosis, and podocyte injury (Huang et al., 2023). However, the specific mechanism by which it improves DKD injuries remained to be explored.

This study aimed to explore and enrich the signaling pathways of scutellarin in treating DKD using network analysis. RNA transcriptome were employed to deepen the understanding of candidate targets in key signaling pathways. Core targets were optimized through various machine learning algorithms. Single-cell transcriptome were utilized to clarify the expression locations and temporal trajectories of core targets, identifying high-expression cell types. Molecular docking and dynamics simulation were applied to predict the binding potential between compounds and biological macromolecules. Finally, cell experiments were conducted to validate the regulatory effects of scutellarin on the molecular targets.

## 2 Materials and methods

### 2.1 Network analysis

Following the suggestions for network analysis methods (Zhang et al., 2023; Nogales et al., 2022), molecular targets of scutellarin and disease were sourced from relevant databases (Supplementary Table S1). Active compound datasets were input into the DrugBank and STITCH databases, filtering for confidence ≥0.7 to obtain target enrichment data. In Swisstarget, the 2D and 3D structures of compounds were input to predict compound targets. DKD-related targets were retrieved from the DrugBank and TTD databases using MESH terms. A Venn diagram of Scutellarin-DKD common targets was created, and the intersecting target dataset was imported into the STRING database to obtain a protein target interaction network (PPI). After calculating key nodes and clusters, the core target interaction map was generated using Cytoscape software. Target clusters were imported into R Studio, with a P-value cutoff of 0.05, minimum overlap of 3, and minimum enrichment of 1.5. Gene Ontology (GO) annotation was performed using the “org.Hs.e.g.,.db” package, categorizing results into Biological Process (BP), Cellular compound (CC), and Molecular Function (MF) (Ding et al., 2018). KEGG analysis was conducted using the “enrichKEGG” package, with the top thirty genes individually subjected to KEGG enrichment (Chen et al., 2017).

### 2.2 RNA transcriptome analysis

The gene expression datasets included GSE99339, GSE30529, GSE96804, GSE104954, and GSE30528. Specimens for GSE96804 and GSE30528 were derived from isolated glomerular tissue of DKD patients, while GSE30529 and GSE104954 specimens were from isolated tubular tissue of DKD patients. GSE99339 was sourced from kidney tissue of DKD patients. Detailed platform and annotation information for these datasets were provided in Supplementary Table S2. The R package “sva” was used to merge these data cohorts and address batch effects, with the ComBat function used for batch effect adjustment. Differential gene expression analysis was performed using the “limma” package, with genes showing log2FC >0.5 and P-value <0.05 designated as differentially expressed genes (DEGs) (Zhou et al., 2023).

Weighted Gene Co-expression Network Analysis (WGCNA) was employed to identify gene modules potentially exhibiting differential expression in the disease context. After excluding outlier samples and adjusting the soft threshold, a weighted gene co-expression network was constructed based on disease binary classification variables. The most strongly correlated modules were intersected with the original DEG dataset, hypoxia and apoptosis geneset (Supplementary Table S3), to refine the gene set selection.

### 2.3 The filter of geneset based on 8 machine learning algorism

8 machine learning algorithms were employed to repeatedly learn the dataset and identify hub genes. The algorithms included Least Absolute Shrinkage and Selection Operator (LASSO) regression, Support Vector Machine Recursive Feature Elimination (SVM-RFE), Regularized Random Forest (RRF), Stepwise Regression, All Possible Regression, Best Subset Regression, Extreme Gradient Boosting (XGBoost) regression, and SHapley Additive exPlanations (SHAP) for XGBoost regression.

For LASSO, the automatically output values were selected and ranked according to the correlation coefficients (Courtois et al., 2021). SVM-RFE selected feature variables consistent with the number of gene sets, used 5-fold cross-validation, and repeated the learning process 3 times. The model’s root mean square error (RMSE) after cross-validation was used as the evaluation metric to output the best gene set, ranked by importance (Sanz et al., 2018). RRF selected the number of option trees that produced the minimum error rate. Model accuracy after cross-validation served as the evaluation metric, with all variables ranked according to Mean Decrease Accuracy and Mean Decrease Gini (Yi et al., 2022). Stepwise Regression initially added features through forward selection, then removed the least contributing features one at a time, outputting the best-fit features based on an acceptable collinearity level (K < 100) (Hauck and Miike, 1991).

All Possible Regression and Best Subset Regression aimed to identify the subset yielding the best predictive results for DKD outcomes, selecting the subset with the highest R^2 value and the lowest MSE, Cp, or AIC value (Salt et al., 2007). The XGBoost model was built using logistic regression, with nfeatures set to 8, niter to 50, and nrounds to 10 (Liang et al., 2021). The DALEX package’s explain function was used for interpretation, with each row representing a feature. In SHAP for XGBoost, the model calculated the SHAP value for each feature of each sample. The contribution of each gene feature to DKD outcomes was represented by the color intensity and size of the bar graph. The top half-ranked genes were output as the best feature gene set (Tarabanis et al., 2023). Genes identified by all machine learning algorithms were designated as hub genes. The DKD composite datasets of GSE47185 and GSE218344 would be used as the validation set. The receiver operating characteristic (ROC) analysis for these hub genes was executed. Area under the Curve (AUC) values exceeding 0.65 were deemed to exhibit commendable diagnostic efficacy (Deng et al., 2022).

### 2.4 Single cell transcriptome analysis

Data from eight samples in the GSE195460 dataset and six samples in the GSE131882 dataset were combined, resulting in six DKD kidney tissues and eight control kidney tissues (non-tumor tissues from nephrectomy patients). Detailed information was provided in Supplementary Table S2. The “Seurat” package was used for preprocessing (Hoffmann et al., 1999). After cell removal, batch correction, feature selection, and high-variable gene enrichment for quality control, data normalization and PCA clustering were performed. The appropriate PC value was selected based on the Elbowplot curve fitting, resulting in different colored modules for clustering. Cell annotation information was obtained from the top three cytokines in the CellMarker 2.0 online database and annotated factors for DKD in the K.I.T database. The hub genes (HIF-1α, CASP3) were included in the clustering map to explore the main cell types with high expression of these genes. Cells expressing both HIF-1α and CASP3, with significant differences compared to the control group (log2FC >0.5 and P < 0.05), were selected for differentiation trajectory and dynamic gene expression analysis.

### 2.5 Molecular docking and dynamics simulation

The 3D structure of the HIF-1α protein was downloaded from the Protein Data Bank (PDB). The crystal structure was ensured to contain a complete pocket, excluding any structures with missing residues. A resolution of <2Å and similarity of the crystal structure to the ligand were set as criteria. The protein structure was processed using Pymol by removing water molecules and ligands, adding hydrogen atoms, calculating charge numbers, and determining rigidity. The 3D structures of the scutellarin was downloaded from PubChem. AutoDock software was used for docking, with the algorithm set to genetic search and output set to local. A two-stage blind-docking process was performed (Hand et al., 2004). In Stage-1, a 126 Å^3^ box (0.375 Å grid) centered on 1H2K chain A (21.3, 4.7, 15.9 Å) sampled the entire protein plus 10 Å cushion. The 200 lowest-energy poses were clustered (2.0 Å RMSD), and a focused 60 Å^3^ box centered on the centroid of the top cluster (residues 495–530 and 775–803) was used for refinement. GA parameters: population 150, 2.5 × 10^7^ evaluations, 27,000 generations, crossover 0.8, mutation 0.02, elitism 1, and Solis-Wets local search every 300 generations (300 steps, ρ = 1.0). Poses were rescored with AutoDock 4.2 and the top pose energy-minimized (conjugate-gradient, 200 steps, 0.02 Å convergence) before acceptance. Re-analysis of blind docking was performed on CB-Dock2 using its curvature-based cavity-detection algorithm (Liu et al., 2022); candidate sites were ranked by Vina score. Concurrently, high-affinity pockets of HIF-1α were identified with P2Rank’s physics-driven scoring, and the binding precision of scutellarin was predicted with a Random Forest model (Polák et al., 2025). Molecular dynamics simulations assessed the flexibility of the protein–ligand complex; iMODS calculated per-residue motion vectors and global dynamics (López-Blanco et al., 2014). System-wide deformability, thermal fluctuations, positional uncertainty, eigenvalues, and residue–residue cross-correlations were evaluated to quantify the drug–target binding potential.

### 2.6 Cell culture

Mouse inner medulla collecting tubular cells (mIMCD-3) were selected as the representative CD-PC commercial cell line for culture (Zheng et al., 2022). Hypoxia modeling was performed in a hypoxia workstation (5% CO2, 1% O2). Since HIF-1α is activated under hypoxia but inhibited by high glucose, four groups were typically set up: N5 (normoxia, 5 mM normal glucose medium), H5 (hypoxia, 5 mM normal glucose medium), H30 (hypoxia, 30 mM high glucose medium), and H30-Scu (hypoxia, 30 mM high glucose medium with 20 μM scutellarin). When necessary, a positive control group H30-DMOG (hypoxia, 30 mM high glucose medium with DMOG, a HIF-1α stabilizer) was included (Fine and Norman, 2008). Cells were passaged every 3 days when 80% confluent using trypsin-EDTA digestion. Passage numbers P5-P10 were preferred, and new cell lines were purchased after 5–10 passages. The details was shown in Table 1; Supplementary Figures S1–S7.

**TABLE 1:** Cell culture system in different experiment.

Experiment	Vessel size	Number of cells	Culture medium	Treatment time
MTT Assay	96-well plate	10,000 per well	RPMI without phenol red	48-h scutellarin intervention after cell seeding
TUNEL Assay	6-well plate	62,500 per well	1.5 mL per well	24-h hypoxic treatment in hypoxic workstation after compound addition
Caspase-Glo^®^ 3/7 Assay	96-well plate	1,500 per well	100ul per well	24-h hypoxic treatment in hypoxic chamber after compound addition
Electron Paramagnetic Resonance Assay	6-well plate	62,500 per well	1.5 mL per well	24-h hypoxic treatment in hypoxic workstation after compound addition
MitoSox Flow Cytometry Assay	6-well plate	62,500 per well	1.5 mL per well	24-h hypoxic treatment in hypoxic workstation after compound addition
HIF1a Transcriptional Activity Assay	12-well plate	25,000 per well	1 mL per well	44-h high-glucose treatment and 24-h hypoxic treatment after compound addition
Nuclear Protein Extraction and Western blot Assay	15 cm dish	1,000,000 per dish	18 mL per dish	6-h hypoxic treatment in hypoxic workstation after compound addition
Immunofluorescence Assay	6-well plate	62,500 per well	1.5 mL per well	6-h hypoxic treatment in hypoxic workstation after compound addition
RNA Extraction, Purification, Reverse Transcription and Amplification	6-well plate	62,500 per well	1.5 mL per well	44-h high-glucose treatment and 24-h hypoxic treatment after compound addition

### 2.7 Molecular experiment

The MTT assay was used to determine the optimal concentrations of scutellarin for mIMCD-3 proliferation. To explore and verify the regulatory effects of these compounds on the hub gene HIF-1α, an HRE-driven luciferase reporter system was used to assess their impact on HIF-1α transcriptional activity and the CTAD/NTAD transcriptional domains of HIF-1α. The plasmids encoding HRE-luciferase and renilla were co-transfected with lipofectamine reagent. The plasmid ratio was 0.12 g pT81/HRE-luc, 0.04 g RLTK/Renilla-luc, 0.24 g CMX vector. In TAD driven luciferase reporting experiments, cells were transfected with plasmids encoding NTAD, CTAD, GAL4-Luciferase, and Renilla. The plasmid ratio was 0.02 g NTAD or CTAD, 0.08 g GAL4-luc, 0.04 g RLTK/Renilla-luc, 0.26 g of CMX vector. Immunofluorescence was employed to qualitatively detect the effects of the compounds on HIF-1α protein expression, while Western blot quantified the effects on HIF-1α nuclear and total protein expression.

At the downstream target genes and phenotypic levels of HIF-1α, RT-PCR was used to measure the impact of the compounds on target genes miR210. Electron paramagnetic resonance (EPR) and MitoSox flow cytometry assessed the effects of the compounds on mitochondrial reactive oxygen species (ROS) release under high glucose and hypoxic conditions. TUNEL staining and Caspase-3/7 activity chemiluminescence assays were conducted to investigate the compounds’ effects on cell apoptosis phenotypes under high glucose and hypoxic conditions. Specific information on materials and equipment were shown in Table 2. The additional information of materials were shown in Supplementary Table S4.

**TABLE 2:** Operation methods in molecular experiments.

Experiment	Equipment	Materials	Operating procedure
MTT Assay	Multiskan SkyHigh Microplate Spectrophotometer	Vybrant^®^ MTT Cell Proliferation Assay Kit Roswell Park Memorial Institute (RPMI) 1640 medium	Add 10 µL of MTT solution to 100 µL of the culture medium as a negative control. After incubation, add 100 µL of SDS-HCl solution and read the absorbance at 570 nm wavelength
TUNEL Assay	Leica dm3000 led Microscope with shutter	*In Situ* Cell Death Detection Kit, POD Triton X-100 3% H2O2 in methanol DNase I, RNase-free Prolong Gold Antifade Mountant with DAPI	Place the slide in contact with TUNEL the reagents, incubate, then wash. Using a fluorescence microscope, induce the emission of green light in the 515–565 nm wavelength range at 450–500 nm
Caspase-Glo^®^ 3/7 Assay	GloMax	Caspase-Glo 3/7 assay (10 mL)	Mix the Caspase-Glo 3/7 reagent, and use the GloMax luminescence instrument (Promega) to detect the luminescence values of Caspase-3/7
Electron Paramagnetic Resonance Assay	Benchtop EPR with the Bruker e-scan	Krebs-Henseleit buffer; mito-TEMPO-H; antimycin A	KHB buffer was used to wash the cells, and then 5.3 μL of 9.5 mM mito-TEMPO-H solution was added. The sample was then loaded into a capillary tube and rapidly frozen in liquid nitrogen. The EPR acquisition parameters were set as default
MitoSox Flow Cytometry Assay	CyAn™ ADP Analyzer by Beckman Coulter Life Sciences	MACS BSA Stock Solution AutoMACS Rinsing Solution MitoSOX Red Mitochondrial Superoxide Indicator	Add 2.0 µL of 5 mM MitoSox stock solution. Incubate, wash, detach the cells, centrifuge, resuspend in FACS buffer. Run the flow cytometer. Analyze using FlowJo software. The mitochondrial ROS level is expressed as a percentage of MitoSOX Red fluorescence intensity
HIF1a Transcriptional Activity Assay	Hypoxia Workstation INVIVO2 (Ruskinn)	Dual-Luciferase Reporter Assay System Lipofectamine™ 3000 Transfection Reagent ZymoPURE II Plasmid; Maxiprep Kit	The plasmids encoding HRE-Luciferase and Renilla were co-transfected into cells using Lipofectamine reagent. After ultrasonic lysis, a fluorescence reaction was conducted, and the optical measurement was used to obtain the activity value of luciferase/Renilla, which represents the nuclear transcriptional activity of the HIF1a transcription factor
Nuclear Protein Extraction and Western blot Assay	Glass Dounce homogenizer Slide-A-Lyzer™ MINI Dialysis Device, 7K MWCO	anti-HIF-1alpha anti-Histone H3 IRDye 800 goat anti-rabbit Secondary Antibody NuPAGE™ LDS Sample Buffer (4X) DTT (dithiothreitol) Halt™ Protease and Phosphatase Inhibitor Cocktail (100X)	After adding the hypotonic buffer, the cell suspension is transferred to the Dounce B type homogenizer for separation and extraction of the cell nuclei. The dialysis tube membrane is rinsed with deionized water, and the protein concentration is measured by the Bradford method. Then, protein loading and electrophoresis are completed
Immunofluorescence Assay	Leica dm3000 led Microscope with shutter	4% formaldehyde Goat anti-Rabbit Alexa 594	Stabilize the cells in PBS containing 0.1% Triton-X100 for 10 min. After washing with PBS, block the cells with PBS containing 5% bovine serum albumin. After incubating with antibodies, mount the coverslips onto slides using DAPI. Take fluorescence images using a Leica DM3000 LED fluorescence microscope
RNA Extraction, Purification, Reverse Transcription and Amplification	MicroAmp™ Optical 384-Well Reaction Plate with Barcode	NucleoSpin miRNA kit Taqman Advanced miRNA Assays;	After cell lysis, the samples were subjected to chromatography, purification, precipitation and elution according to the NucleoSpin^®^ instructions. Using the micro RNA reverse transcription kit. Quantitative RT-PCR was performed on the 7300 or 7900 Real-Time PCR system, and the expression level of the miR191 gene was used as the control

## 3 Result

### 3.1 Network analysis showed that scutellarin might treat DKD by modulating hypoxic responses and apoptotic pathways

**FIGURE 1:**
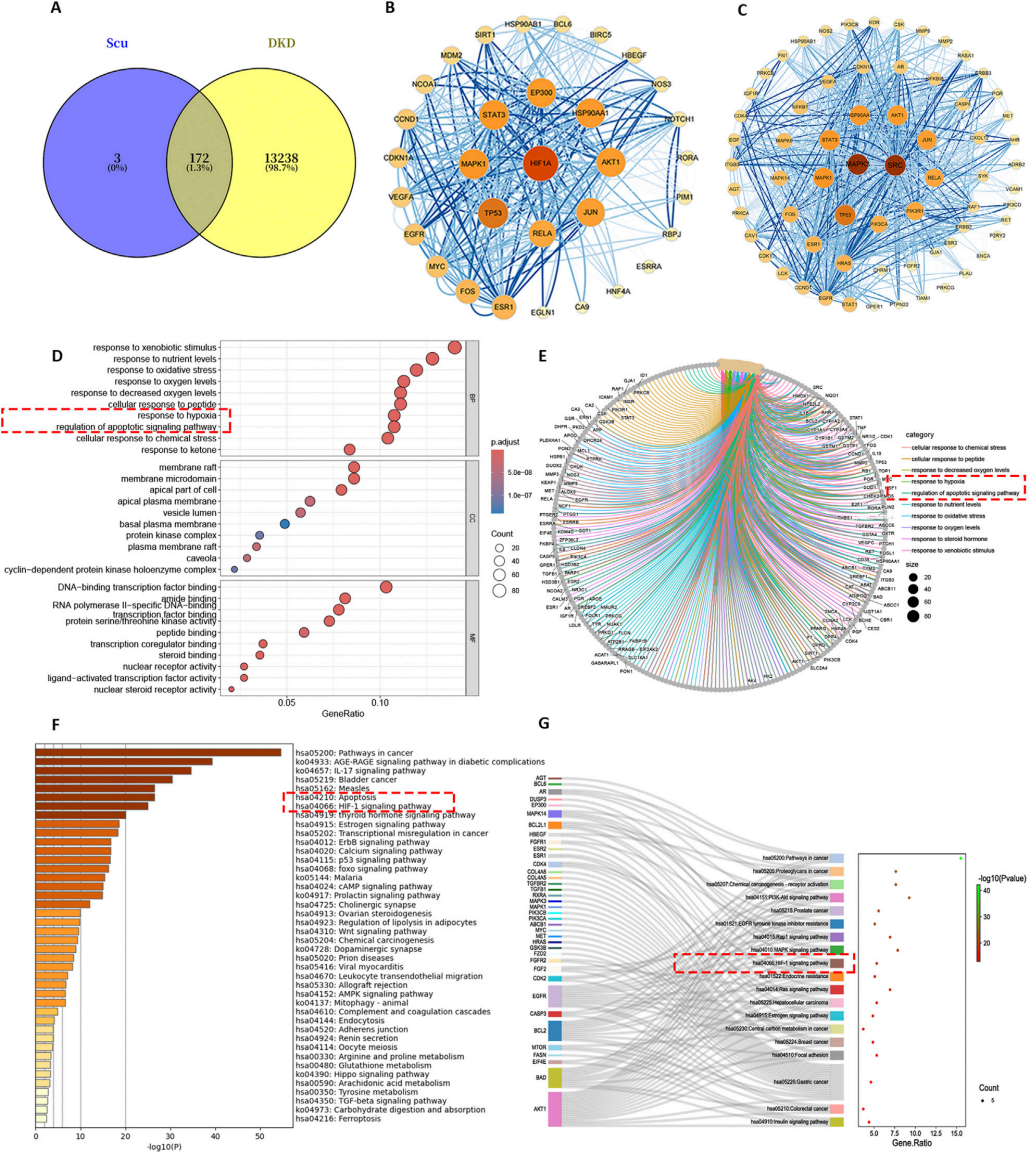
The result of network analysis. **(A)** Target intersection of Scutellarin and DKD. **(B)** Core target interaction map of HIF-1α. **(C)** Core target interaction map of MAPK3. **(D,E)** GO functional annotation. **(F,G)** KEGG functional annotation.

The scutellarin was found to target a total of 175 biological targets. The intersection with DKD disease resulted in 172 active targets (Figure 1A). Two key clusters naturally formed: the HIF-1α cluster (Figure 1B) and the MAPK3 cluster (Figure 1C). The stronger the interaction, the larger the target circle. GO analysis (Figures 1D,E) revealed that key biological processes annotated for the targets include response to hypoxia, response to decreased oxygen, and regulation of apoptotic signaling. KEGG analysis (Figures 1F,G) showed that key pathways annotated for the targets include the HIF-1 signaling pathway and apoptosis. So the response to hypoxia and apoptotic pathways may be key mechanisms through which the scutellarin treated DKD.

### 3.2 DKD bulk RNA revealed eight differentially expressed genes related to hypoxia response and apoptosis pathway

**FIGURE 2:**
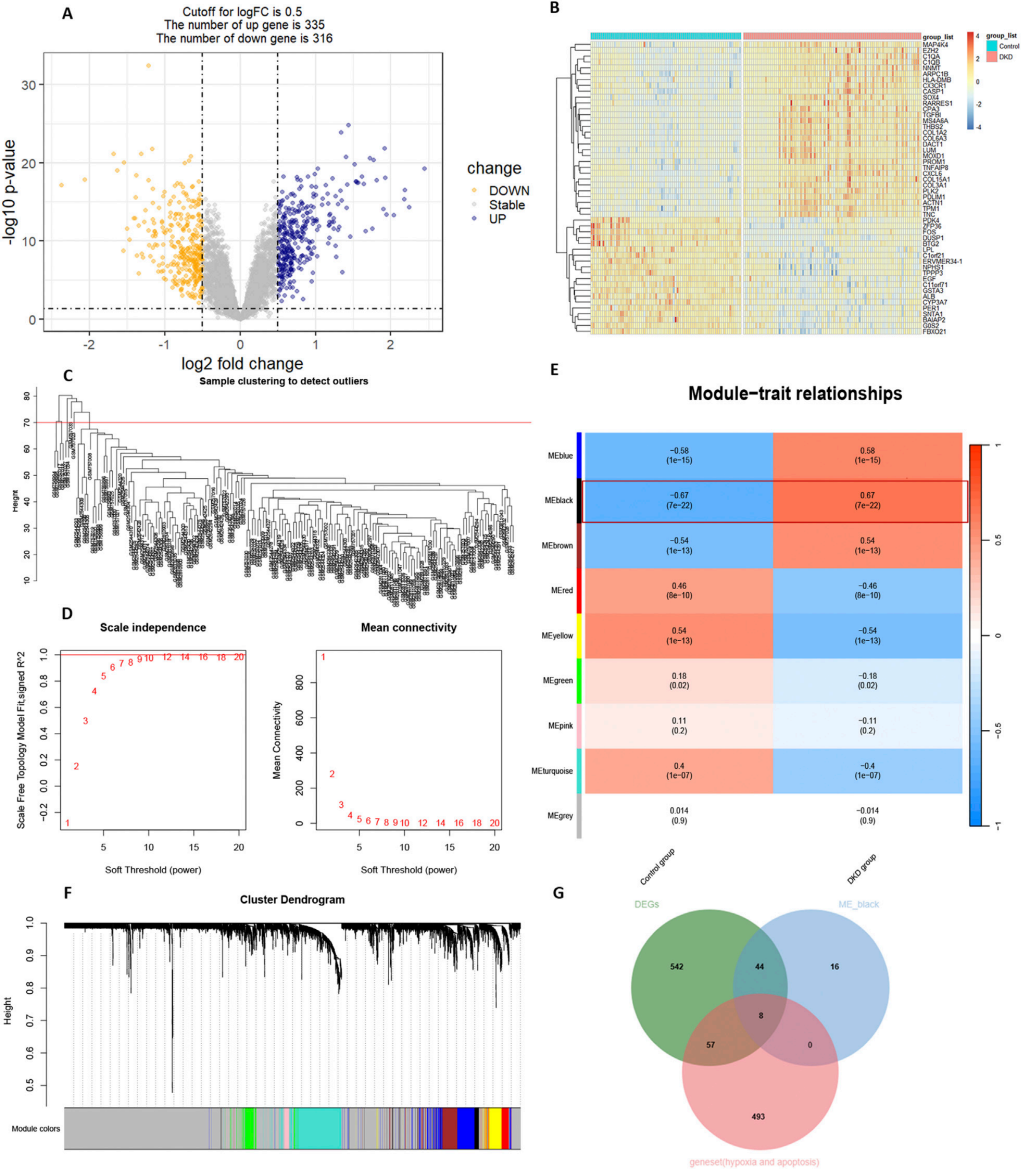
Bulk RNA transcriptome analysis. **(A)** Volcano plot of differentially expressed genes (DEGs). **(B)** Heatmap of the top 50 DEGs. **(C)** Clustertree by WGCNA. **(D)** Soft threshold setting. **(E)** Correlation analysis between gene modules and diseases. **(F)** Cluster dendrogram of gene modules. **(G)** Intersection of strongly correlated gene modules with DEGs, hypoxia and apoptosis geneset.

The results of the DKD bulk RNA transcriptome analysis were shown in Figure 2. Data preprocessing was shown in Supplementary Figure S8. The volcano plot indicated 335 upregulated and 316 downregulated genes (Figure 2A). Among the top fifty differentially expressed genes, thirty were significantly upregulated, while twenty were downregulated (Figure 2B). WGCNA identified eight outlier samples (Figure 2C). After excluding these samples, the curve smoothed at a soft threshold of 14 (Figure 2D). Based on this threshold, KNN clustering revealed nine gene modules, with the ME-black module showing the strongest association with the DKD variable (P = 7e-12, |Q| = 0.67) (Figure 2E). The ME-green module contained the most gene variables (Figure 2F). The intersection of the ME-black module, DEGs, hypoxia, and apoptosis gene sets identified eight key genes: CASP3, HS3ST1, CLU, TUBA1A, CASP4, PFKP, TIMP1, and HIF-1α.

### 3.3 Machine learning identified HIF-1α and CASP3 as hub genes for DKD

**FIGURE 3:**
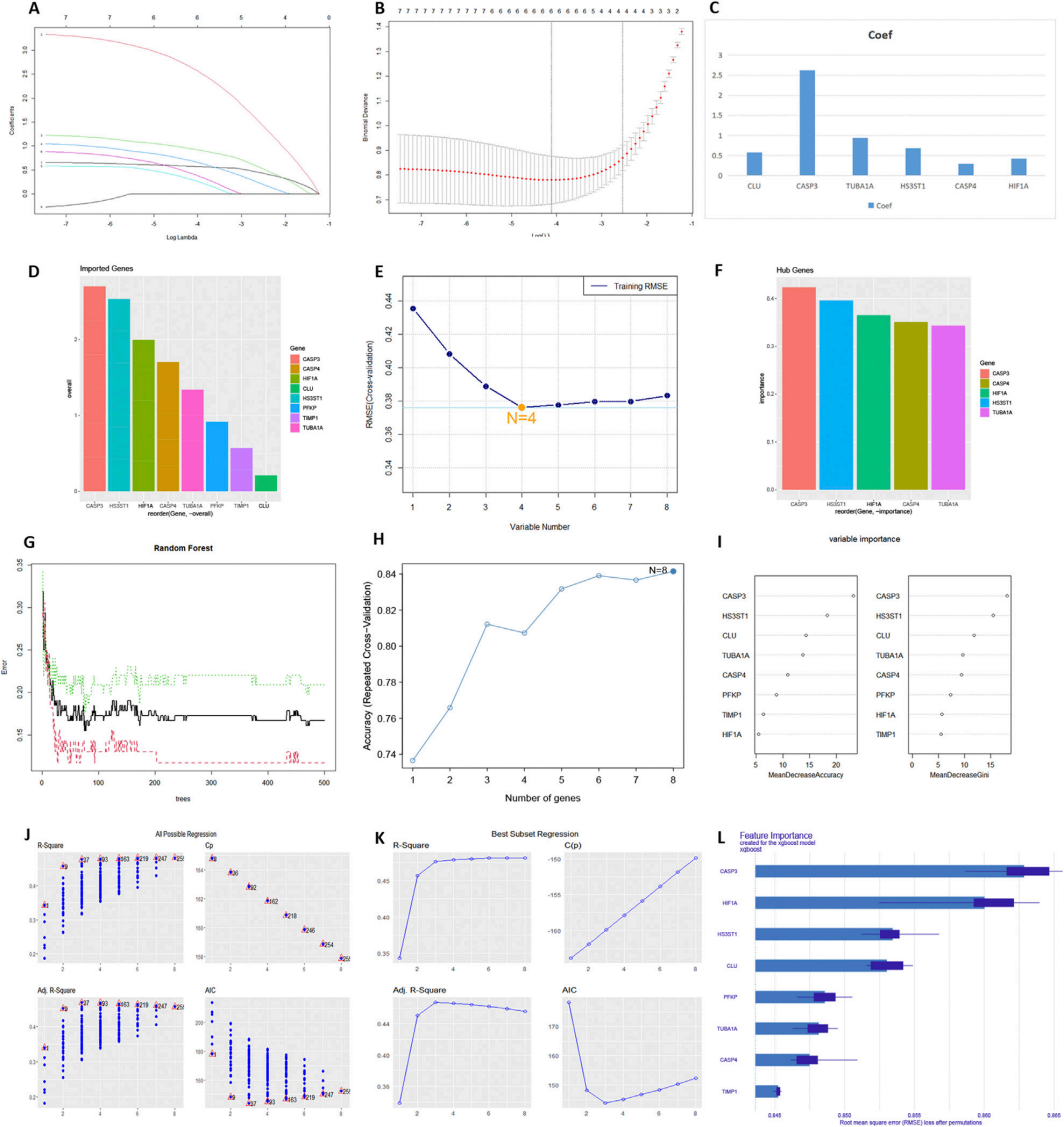
The output of multiple machine learning algorithms. **(A–C)** LASSO regression. **(D–F)** SVM-RFE. **(G–I)** RRF. **(J)** All Possible Regression. **(K)** Best Subset Regression. **(L)** XGBoost regression.

The results of the machine learning analysis were shown in Figure 3. In the LASSO regression, most genes were positively correlated with DKD (Figure 3A). Six genes were selected to form the predictive model (Figure 3B), ranked by correlation coefficient: CASP3, TUBA1A, HS3ST1, CLU, CASP4, and HIF-1α (Figure 3C). In the SVM-RFE analysis, all input genes were ranked from CASP3 to TUBA1A (Figure 3D). The model had the lowest RMSE when the number of variables was four (Figure 3E), with the hub genes ranked by importance as CASP3, CASP4, HIF-1α, and HS3ST1 (Figure 3F). The RFF analysis used 500 decision trees (Figure 3G) and showed the highest accuracy when the number of variables was eight (Figure 3H). Genes were ranked from CASP3 to TIMP1 (Figure 3I). Both All Possible Regression (Figure 3J) and Best Subset Regression (Figure 3K) indicated the highest predictive validity when n = 8. Stepwise Regression identified CLU, CASP3, TUBA1A, and HIF-1α as key variables in the model (Supplementary Figures S9A,B). XGBoost regression showed the top variables as CASP3, HIF-1α, HS3ST1, CLU, and PFKP (Figure 3L). Each variable’s partial dependence profile was shown in Supplementary Figures S14C. SHAP value analysis ranked the top variables as CASP3, HS3ST1, HIF-1α, PFKP, and CASP4 (Supplementary Figures S9C–E). The intersection of all results indicated that HIF-1α and CASP3 were hub genes (Supplementary Figure S10A). Both genes were significantly upregulated in the DKD patient test set (Supplementary Figure S10B). ROC analysis showed an AUC of 0.726 for CASP3 and 0.652 for HIF-1α in the validation set (Supplementary Figure S10C).

### 3.4 Single-cell transcriptome revealed CD-PC as the primary cell type for hub gene differential expression

**FIGURE 4:**
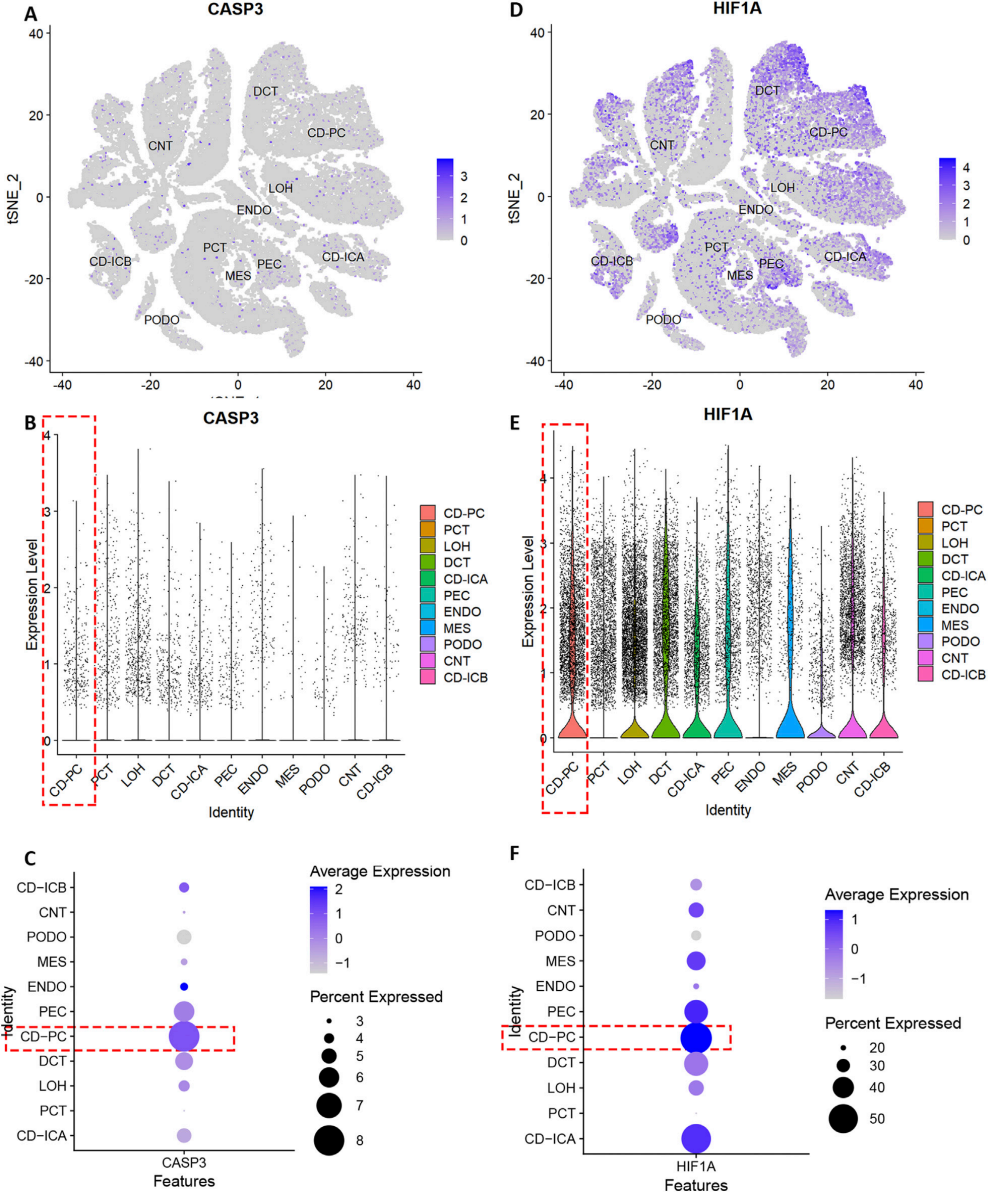
Expression of hub genes in different types of kidney cells. **(A–C)** Distribution and expression of CASP3. **(D–F)** Distribution and expression of HIF-1α.

**FIGURE 5:**
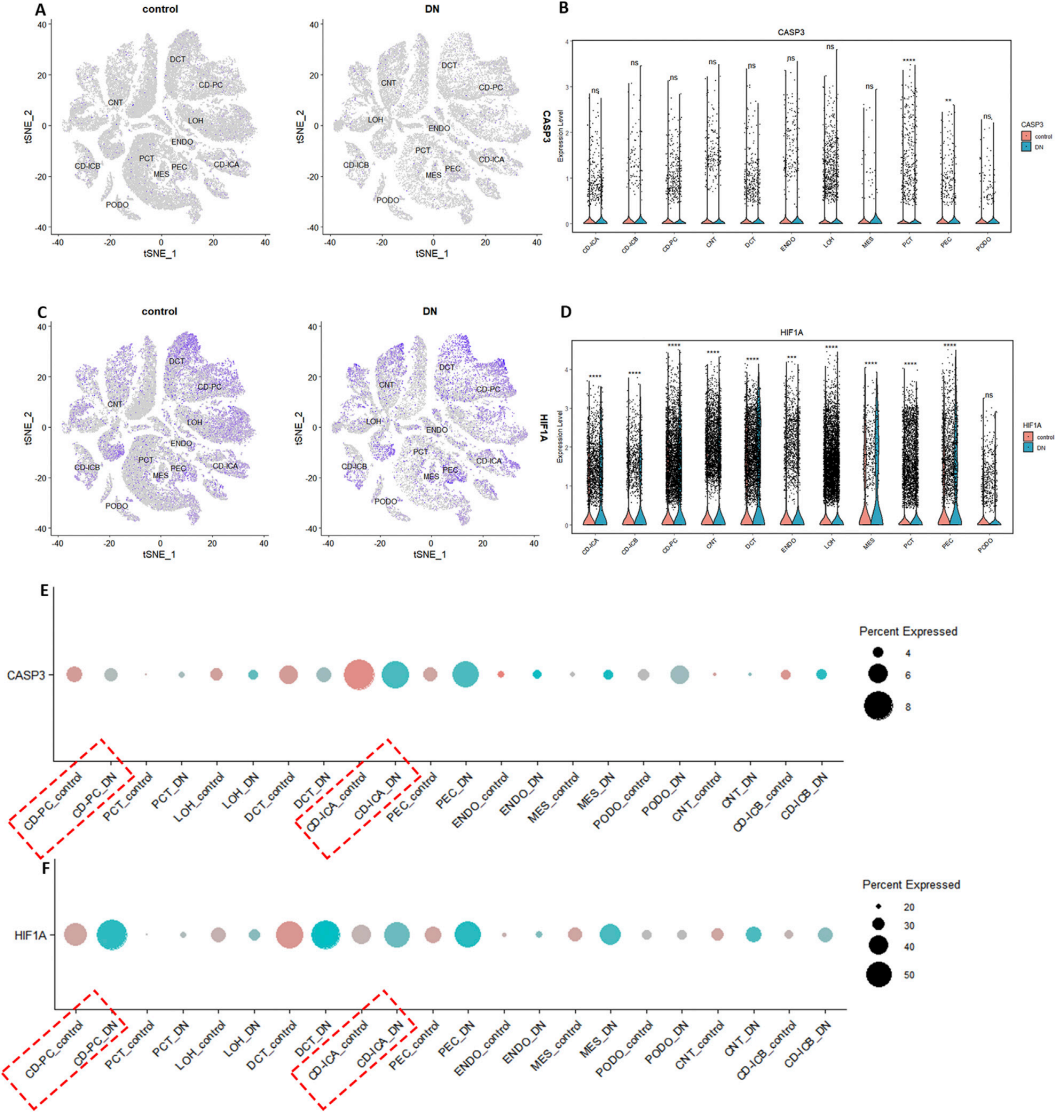
Expression of hub genes in DN single cells. **(A,B,E)** Distribution and expression of CASP3. **(C,D,F)** Distribution and expression of HIF-1α.

The quality control results of the single-cell dataset were shown in Supplementary Figure S11. Low heterogeneity was observed between samples, with no interference from mitochondrial, ribosomal, or erythrocyte RNA. Batch effects were not significant, and cell types were similar across samples. Dimensionality reduction and clustering were performed using 3000 highly variable genes. In the DN group, the combined data were effectively reduced to 14 clusters and 11 cell types: collecting duct-principal cell (CD-PC), proximal convoluted tubular cell (PCT), Loop of Henle cell (LOH), distal convoluted tubular cell (DCT), collecting duct-intercalated cell type A (CD-ICA), glomerular parietal epithelial cell (PEC), endothelial cell (ENDO), mesenchymal cell (MES), podocyte (PODO), connecting tubular cell (CNT), and collecting duct-intercalated cell type B (CD-ICB) (Supplementary Figures S11F,G). CASP3 showed low overall expression and there was a relatively high level of expression in CD-PC cells (Figures 4A–C). HIF-1α had higher overall expression and there was also a relatively high level of expression in CD-PC cells (Figures 4D–F). Compared with normal groups, CASP3 was relatively more distributed in CD-PC and CD-ICA cells in DN samples (Figures 5A,B,E). Compared to the control group, HIF-1α was significantly upregulated in CD-PC cells of DN samples (Figures 5D–F).

All markers in different cell type were shown in Supplementary Figure S12. The differential expression of hub gene and pseudotime analysis of CD-PC was shown in Supplementary Figure S13. HIF-1α expression was highest in moderately differentiated CD-PC cells and lowest in undifferentiated CD-PC cells, indicating a positive correlation between HIF-1α expression and cell differentiation. CASP3 expression was also highest in moderately differentiated PEC cells and lowest in highly differentiated mature CD-PC cells, suggesting a negative correlation between CASP3 expression and cell differentiation. The cell communication analysis of CD-PC was shown in Supplementary Figure S14, in which GDF signalling pathway exerted a crucial part.

### 3.5 Molecular docking indicated potential binding capability of scutellarin with HIF-1α

**FIGURE 6:**
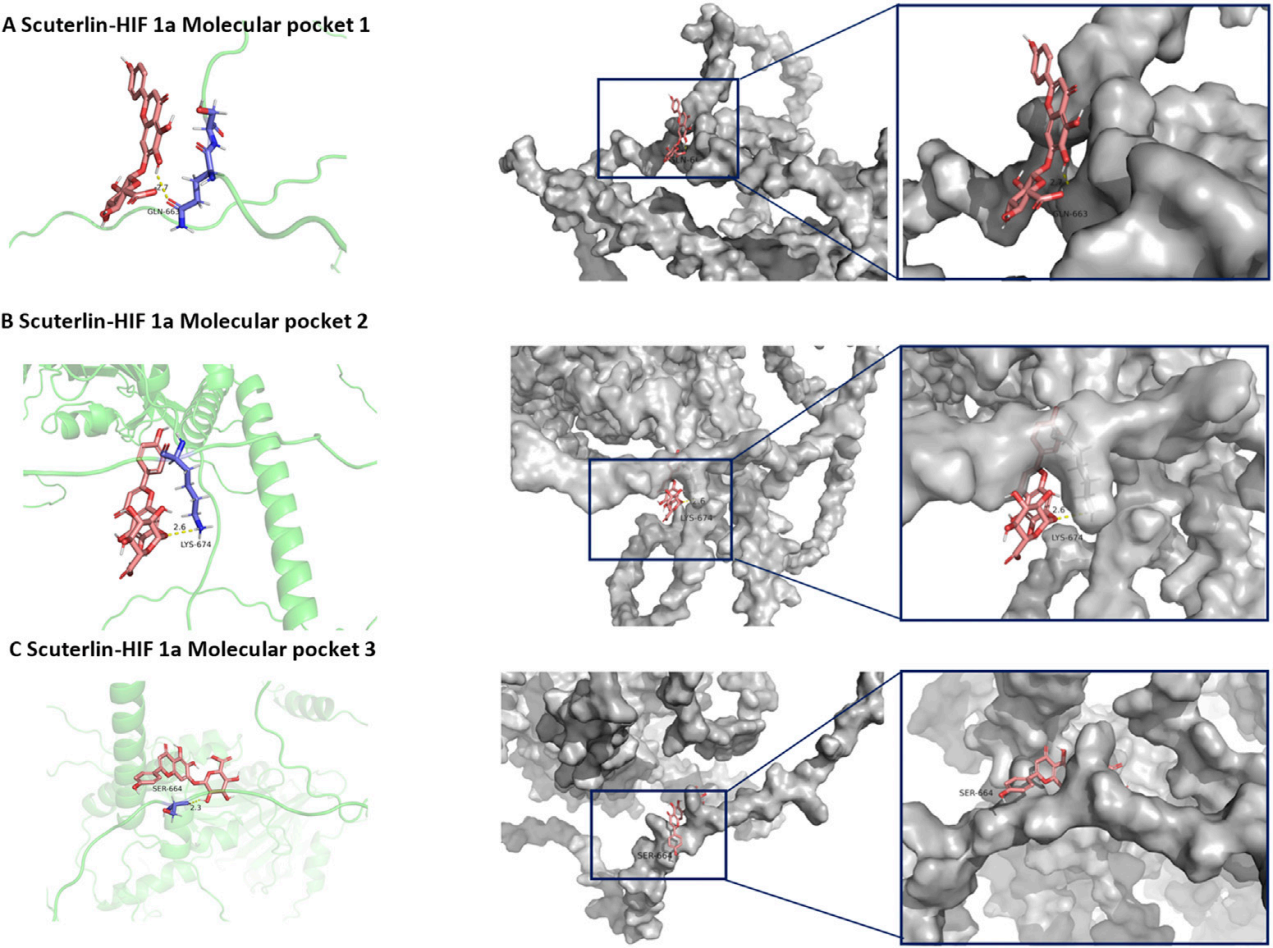
Molecular Docking Results of scutellarin with HIF-1α. **(A)** The first docking locus. **(B)** The second docking locus. **(C)** the third docking locus.

**FIGURE 7:**
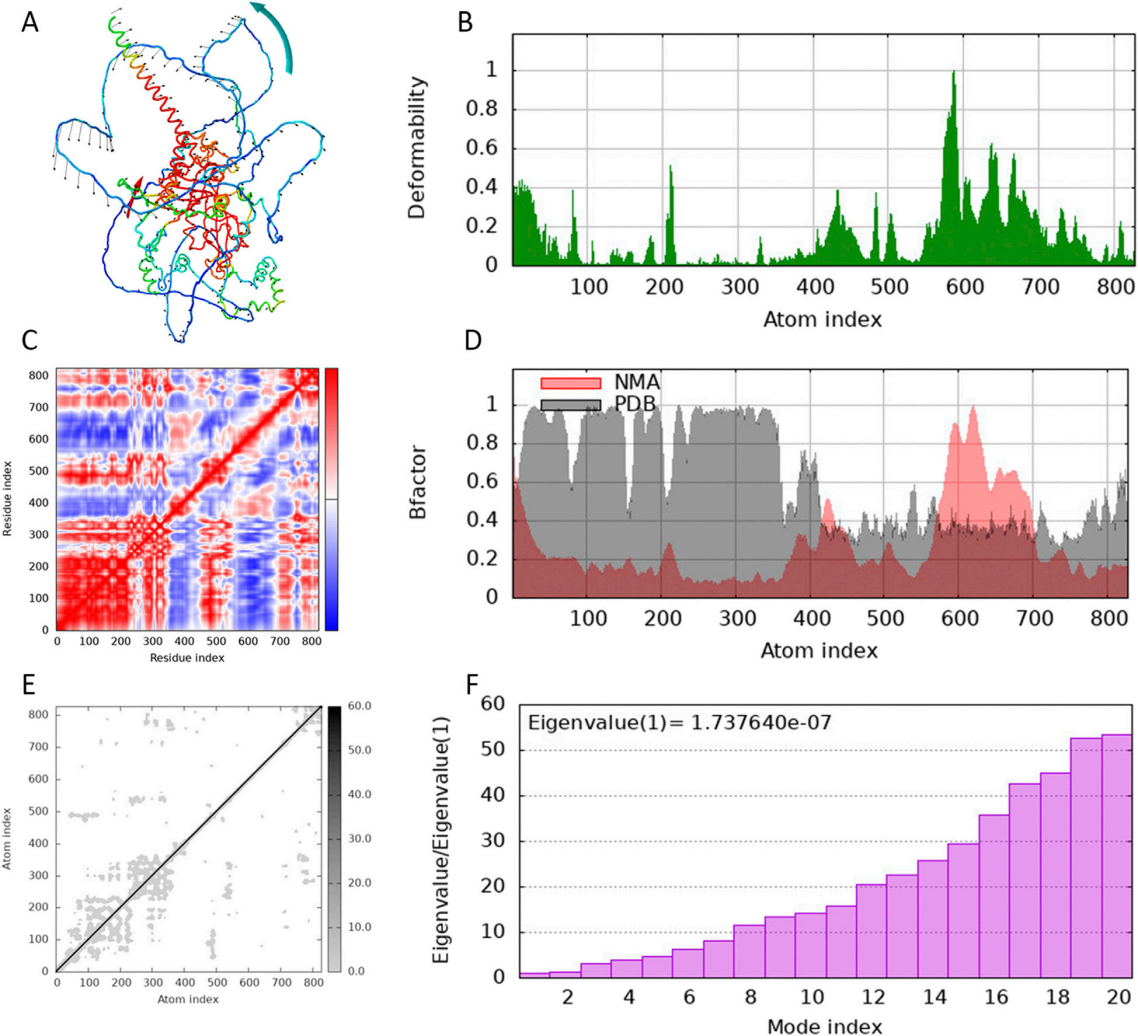
Molecular dynamics simulation of scutellarin with HIF-1α. **(A)** The amplitude of amino acid movement. **(B)** The deformability map of amino acid residues. **(C)** The covariance map of movements among amino acid residues. **(D)** The B factor diagram of the thermal vibration amplitude. **(E)** Elastic network model. **(F)** Eigenvalue of protein conformational change.

The molecular docking energies and specific binding sites of the scutellarin with HIF-1α based on Autodock were listed in Supplementary Table S5. The first docking locus involved the protein residue GLN-164 (Figure 6A), with a mixed hydrogen bond energy of −5.6, with ligand efficiency equal to −0.27, Intermol energy equal to −4.31 and electrostatic energy equal to −0.15. The second docking locus involved the protein residues LYS-674 (Figure 6B), with a mixed hydrogen bond energy of −5.22, with ligand efficiency equal to −0.21, Intermol energy equal to −3.12 and electrostatic energy equal to −0.05. The third docking locus involved the protein residues SER-664 (Figure 6C), with a mixed hydrogen bond energy of −5.17, with ligand efficiency equal to −0.21, Intermol energy equal to −3.06 and electrostatic energy equal to −0.01, all showing a clear nested pocket structure on the molecular surface. Blind molecular docking based on CB-DOCK2 also revealed that scutellarin has 5 binding pockets with protein HIF-1α, with the lowest score being −6.5 and the highest score being −8.3 (Supplementary Figure S15; Supplementary Table S6). Targeted molecular docking based on P2RANK platform revealed that scutellarin could embed in the natural pocket and binding hot spot formed by the HIF-1α protein, with the maximum score of −13.07 (Supplementary Figure S16; Supplementary Table S7). The molecular dynamics simulation of scutellarin with HIF-1α was shown in Figure 7. A global helical motion was observed (Figure 7A). The binding interface displayed moderate flexibility, with peak deformability localized at atom indices 400–600 (Figure 7B). Consistent directional motion (red covariance) within this region indicated that these residues formed a cooperative functional unit, ensuring stable ligand binding (Figure 7C). B-factors fluctuated within the same 400–600 range without perturbing the intrinsic protein dynamics (Figure 7D). The lighter gray elastic-network springs in this zone denoted reduced stiffness (Figure 7E). The dominant eigenvalues appeared at model indices 14–18, reflecting the primary conformational modes of the complex (Figure 7F).

### 3.6 Scutellarin reduced apoptosis and ROS in collecting duct cells and upregulated HIF-1α transcriptional activity

**FIGURE 8:**
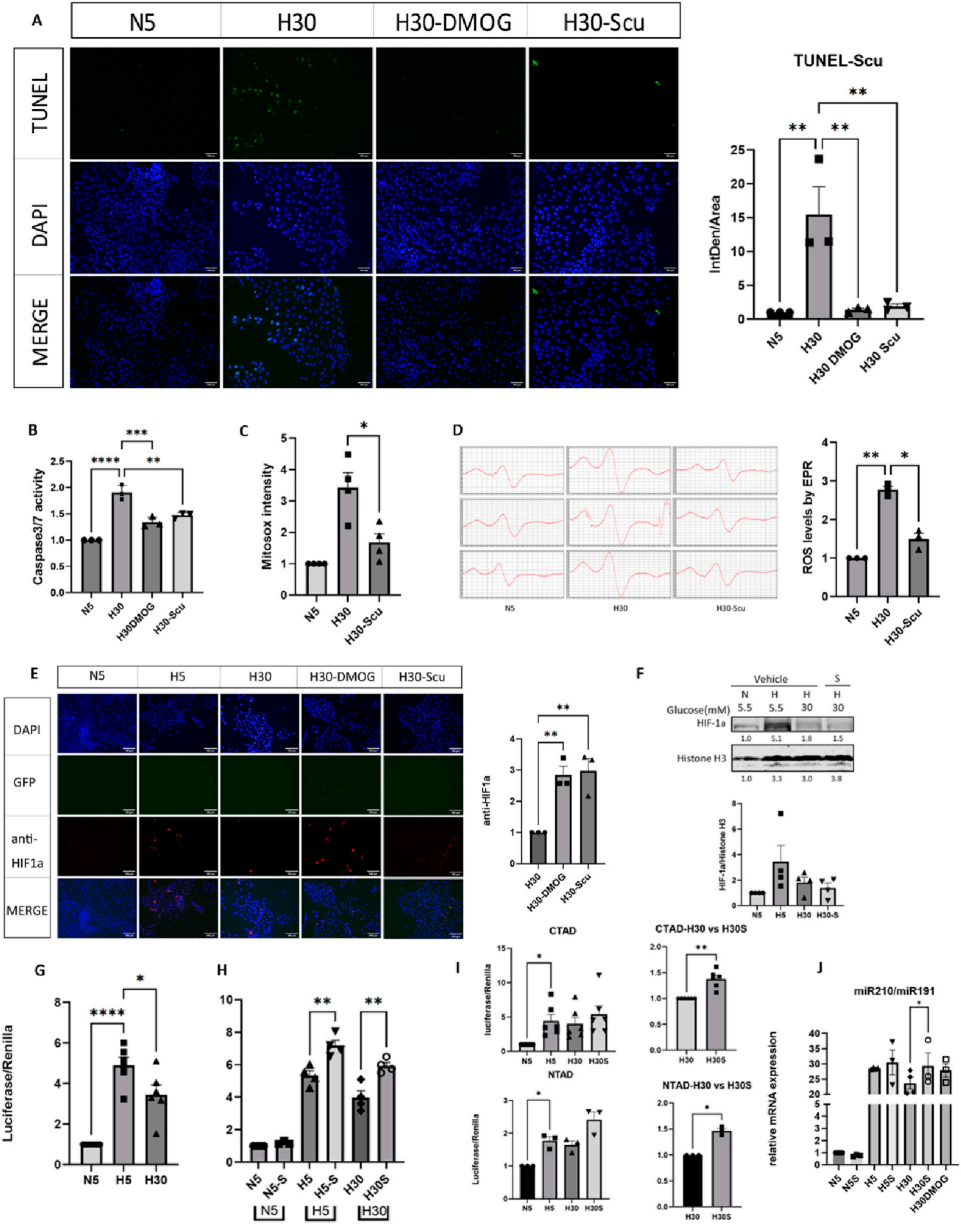
Effects of scutellarin on mitochondrial ROS release, cell apoptosis and HIF-1α function. N5: Normoxia and normal glucose medium; H5: Hypoxia and normal glucose medium; H30: Hypoxia and high glucose medium; S: Scutellarin; DMOG: dimethyloxallyl glycine, the HIF-1α stabilizer. *P (One-way ANOVA) <0.05, **P (One-way ANOVA) <0.01, ***P (One-way ANOVA) <0.001, ****P (One-way ANOVA) <0.0001. **(A)** The effect of scutellarin on cell apoptosis was detected by TUNEL assay. **(B)** The effect of scutellarin on Caspase-3/7 activity was measured using the Caspase-Glo^®^ 3/7 Assay. **(C)** The effect of scutellarin on mitochondrial ROS release in cells was analyzed by flow cytometry. **(D)** Mitochondrial free radical levels in cells treated with scutellarin were measured using EPR. **(E)** IF for total HIF-1α under the treatment of scutellarin. **(F)** The effect of scutellarin on nuclear HIF-1α protein. **(G)** Luciferase-reporter measurement for the HIF-1α transcriptional activity under N5, H5, H30. **(H)** Luciferase-reporter measurement for the HIF-1α transcriptional activity under the treatment of scutellarin. **(I)** Luciferase-reporter measurement for HIF-1α transcriptional domains CTAD and NTAD under the treatment of scutellarin. **(J)** RT-PCR result of HIF-1α downstream target genes miR210 under the treatment of scutellarin.

The MTT assay indicated that 20 μM scutellarin (P < 0.05) resulted in the highest OD values, which exhibited a peak effect and most beneficial for cell proliferation (Supplementary Figure S17). TUNEL staining showed that fluorescence intensity significantly increased in mIMCD-3 cells under high glucose and hypoxia conditions (H30) (P < 0.01). Treatment with scutellarin significantly reduced the fluorescence signal (P < 0.01) (Figure 8A). Scutellarin also significantly decreased caspase-3/7 activity levels (P < 0.01) (Figure 8B), indicating that scutellarin effectively reduced apoptosis in mIMCD-3 cells under high glucose and hypoxia conditions. After MitoSox staining, fluorescence intensity was significantly higher in the H30 group (P < 0.01), while the H30-Scutellarin group showed a reduction (P < 0.05) ((Figure 8C). EPR analysis revealed that the electron spin resonance (ESR) signal significantly increased in the H30 model group (P < 0.01), and was reduced following treatment of scutellarin (P < 0.05) (Figure 8D).

After transfection with GFP-HIF-1α plasmid and hypoxia induction, a noticeable red fluorescence signal was observed. The H30-Scutellarin, and H30-DMOG groups showed significant increases in fluorescence (P < 0.05), similar to the positive control DMOG (P < 0.05) (Figure 8E). WB results, with Histone H3 as a nuclear protein reference, showed an increase in the H5 group compared to the N5 group, while a decrease in the H30 group compared to the H5 group. However, no significant changes were noted in the H30-Scu group (Figure 8F). The luciferase reporter system demonstrated that with Renilla as a reference, the luciferase intensity in the H5 group was significantly higher than in the N5 group (P < 0.0001), while it was significantly lower in the H30 group compared to the H5 group (P < 0.05) (Figure 8G). This suggested that hypoxia significantly enhanced HIF-1α gene transcription driven by HRE conserved genes, whereas high glucose significantly inhibited hypoxia-induced HIF-1α transcription. The luciferase intensity in the H30-S was significantly higher than in the H30 group (P < 0.05), while H5-S group was higher than H5 group (P < 0.01) (Figure 8H). The luciferase intensity in the H30-S group was significantly higher than in the H30 group for both CTAD and NTAD (P < 0.05) (Figure 8I). The PCR results showed the expression of HIF-1α downstream target gene miR210 was significantly higher in hypoxia while lower in hyperglycemia. The expression of miR210 in H30-S group was higher than that in H30 group (Figure 8J). The original WB banding pattern, the cell flow cytometry gated image, and the PCR amplification plot were shown in Supplementary Figure S17.

## 4 Discussion

This study explored the molecular mechanisms of scutellarin in treating DKD using network analysis, emphasizing the roles of hypoxic response and apoptosis pathways. RNA transcriptome and machine learning identified key gene sets related to DKD outcomes and hypoxic apoptosis. Single-cell transcriptome analysis confirmed the expression patterns and locations of hub genes, identifying the CD-PC cells as the high-expression cell type. *In vitro* experiments demonstrated scutellarin’s regulation of HIF-1α and its protective effect against cell apoptosis.

DKD is characterized by chronic hypoxia. High glucose levels lead to hypoxia in renal cells through several pathological pathways. First, high glucose and hyperperfusion cause glomerular hyperfiltration, increasing tubular reabsorption and oxygen consumption, leading to functional hypoxia in the tubules (Chae et al., 2023). Second, persistent high glucose induces oxidative stress and ferroptosis, damaging mitochondrial function and disrupting the respiratory chain, impairing oxygen synthesis (Wang and Chen, 2022). Third, dysregulated glucose and lipid metabolism causes endothelial dysfunction, impaired vasodilation, increased vasoconstriction, elevated vascular resistance, and reduced renal blood flow and perfusion (Tanaka and Nangaku, 2013). Additionally, increased ATP demand and reduced ATP generation result in ATP deficiency and increased renal QO_2_, making kidney more susceptible to hypoxic injury (Nangaku, 2006; Spencer et al., 2021). The stable expression of HIF-1α is crucial for hypoxia tolerance. HIF-1α is activated under hypoxic conditions (Koyasu et al., 2018). Therefore, its significant upregulation was observed in DKD patients in both RNA and single-cell transcriptome analyses. Under high glucose conditions, HIF-1α nuclear translocation efficiency decreased, degradation accelerated, and transcriptional activity reduced, impairing the hypoxic adaptive response and leading to oxidative stress-related damage (Catrina and Zheng, 2021; Thangarajah et al., 2010). Thus, in cell experiments, both Western blot and HRE-driven luciferase-reporter measurements showed higher levels in H5 compared to N5 and lower levels in H30 compared to H5. The modulating effect of scutellarin on HIF-1α is to antagonize the inhibition effect of high glucose on HIF-1α, thereby reducing ROS release and reducing apoptosis of renal tubule cells.

At the phenotypic level, scutellarin reduced mitochondrial ROS release and apoptosis in mIMCD-3 cells under high glucose and hypoxia. This finding was consistent with previous literature. Wang Z et al. reported that scutellarin promoted JAK/STAT3 activation and inhibited the expression of Bax and caspase-3, reducing ROS production to prevent myocardial ischemia-reperfusion injury (Wang et al., 2016). Yang L et al. Found that scutellarin could improve mitochondrial dysfunction and inhibit apoptosis by stimulating mitophagy, thereby attenuating OGD/R-induced HT22 cells injury (Yang et al., 2024). In tubular cell, scutellarin could increase the expression of antioxidant enzyme HO-1, thereby reducing the level of ROS. As a result, the degree of apoptosis in HK-2 cells incubated under hypoxia/reoxygenation (H/R) conditions was alleviated (Dai et al., 2022). Tubular cell apoptosis is a hallmark of tubular injury in DKD. Mediating HIF-1α to regulate the hypoxic response and alleviate tubular cell apoptosis might be an important mechanism for scutellarin in treating DKD. For HIF-1α pathway, scutellarin significantly upregulated HIF-1α transcriptional activity by activating its domain especially CTAD. It also significantly increased HIF-1α total protein fluorescence expression, similar to the positive control DMOG. This finding aligned with previous research. Bogacz et al. also found that scutellarin upregulated HIF-1α mRNA and thus reducing cell inflammation (Bogacz et al., 2021).

miR-210 was selected as the representative downstream target of HIF-1α. In hypoxic tissues. HIF-1α escapes VHL-mediated degradation, accumulates in the nucleus, and transcriptionally activates a battery of adaptive genes. Among these, miR-210 is consistently and robustly induced: multiple solid tumours, ischaemic heart tissue, and VHL-null renal carcinoma all show strong positive correlations between HIF-1α stabilisation and miR-210 upregulation (Ivan and Huang, 2014). Chromatin-immunoprecipitation and promoter-reporter assays further demonstrate direct binding of the HIF-1α/ARNT heterodimer to a hypoxia-response element located ∼40 bp upstream of the miR-210 precursor (Kulshreshtha et al., 2007a; Kulshreshtha et al., 2007b). Consequently, miR-210 is widely regarded as a faithful downstream transcriptional read-out of HIF-1α activity in both physiological and pathological settings.

However, we found that scutellarin has no significant effect on the degradation of nuclear proteins. Its regulatory role consist in the total protein level of HIF-1α. The stability of HIF-1α nuclear translocation is affected by various environmental factors, and the degradation rate is relatively fast. The scutellarin might regulate the stability of cytoplasmic HIF-1α protein through processes such as enhancing the interaction between HIF-1α and molecular chaperones like heat shock protein 90 (Hsp90), which can prevent the recognition of HIF - 1α by E3 ubiquitin ligases and thus delay its degradation (Isaacs et al., 2002). It might also inhibit the activity of prolyl hydroxylase domain (PHD) enzymes that target HIF-1α for hydroxylation and subsequent proteasomal degradation (Fong and Takeda, 2008). The confirmation of the relevant mechanism still requires further experimental verification.

The novelty of this study resided in its multi-layered strategy: starting from bulk and single-cell transcriptomes, we leveraged state-of-the-art machine-learning frameworks to progressively distill the hypoxia- and apoptosis-centric circuitry underlying DKD This pipeline ultimately nominated collecting-duct principal cells and the HIF-1α protein as the primary targets for *in vitro* interrogation. Unlike previous investigations that often relied on a single read-out, we interrogated scutellarin’s impact on HIF-1α at three complementary levels—nuclear translocation, total protein abundance, and transcriptional output—thereby delineating a nuanced map of how the compound reshaped tubular hypoxia signaling. These findings provided a conceptual scaffold for future mechanism-oriented studies of DKD pharmacotherapy.

Several limitations were acknowledged. First, although *in silico* docking coupled with molecular-dynamics simulations offered mechanistic hypotheses, scutellarin’s polyphenolic backbone rendered it prone to the nonspecific membrane and protein interactions characteristic of Pan-Assay Interference Compounds (PAINS). Consequently, all computational predictions required orthogonal validation by biophysical methods (SPR, ITC) and functional cell-based assays before any pharmacological claim could be asserted; such experiments were planned. Second, our mechanistic evidence remained preliminary: precisely how scutellarin enhanced HIF-1α transcriptional activity while simultaneously curtailing its proteasomal degradation awaited further biochemical dissection.

## 5 Conclusion

Through Network analysis, molecular docking, and single-cell transcriptome analysis, it was found that HIF-1α in the hypoxic response pathway was a key gene for DKD. Scutellarin significantly reduced renal cell apoptosis under high glucose and hypoxia by modulating HIF-1α and reducing mitochondrial ROS release.

## Data Availability

The original contributions presented in the study are included in the article/Supplementary Material, further inquiries can be directed to the corresponding author.
