# Supplementary material for: Scutellarin attenuated tubule cell apoptosis by modulating HIF-1α for the treatment of DKD: the insight integrating network analysis, machine learning and single-cell transcriptome
Source: Front Pharmacol. 2025 Sep 30;16:1656409. doi: 10.3389/fphar.2025.1656409 (PMC12517588; doi:10.3389/fphar.2025.1656409)
Supplement: Supplementary file 1 [file Supplementaryfile1.docx]

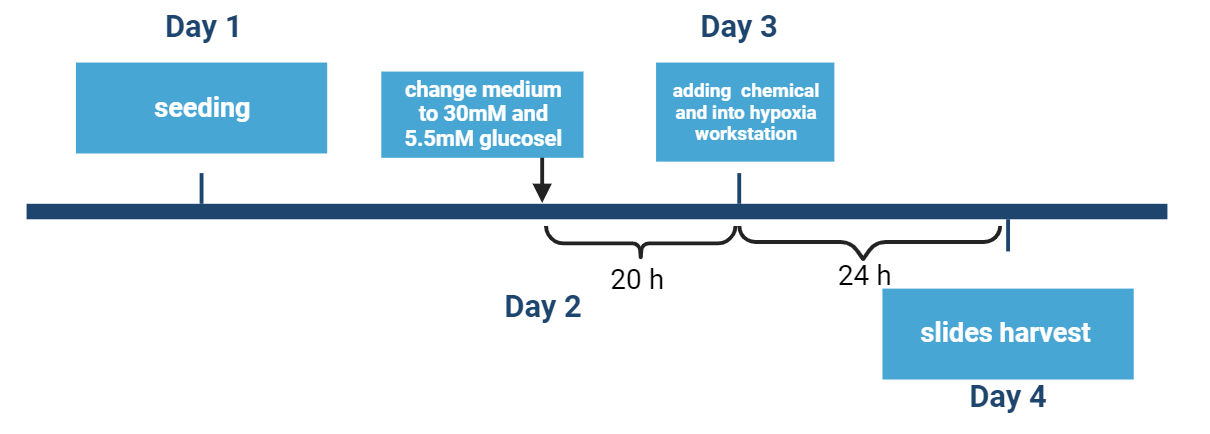


**Supplementary Figure 1** Timeline for TUNEL


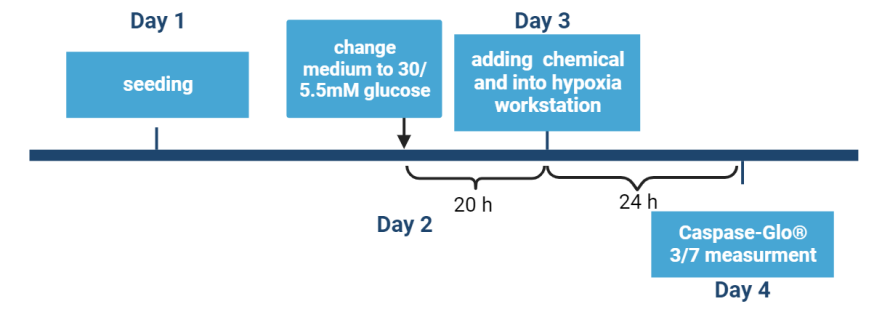


**Supplementary Figure 2** Timeline for Caspase- 3/7 method


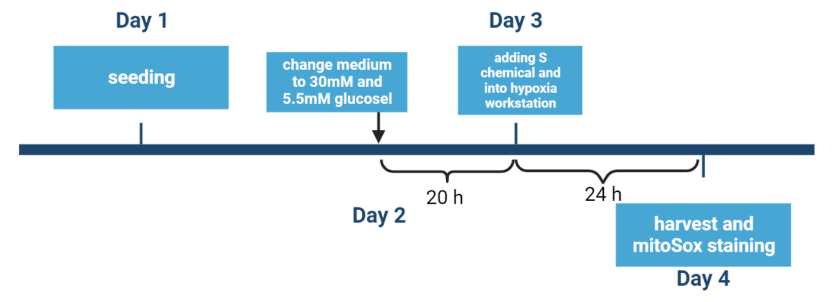


**Supplementary Figure 3** Timeline for MitoSox cytometry


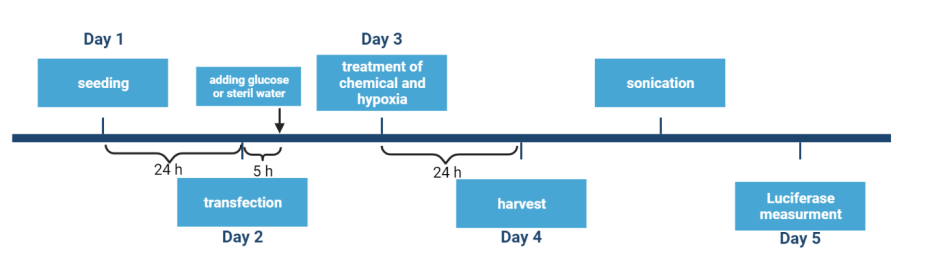


**Supplementary Figure 4** Timeline for HRE-driven luciferase reporter system


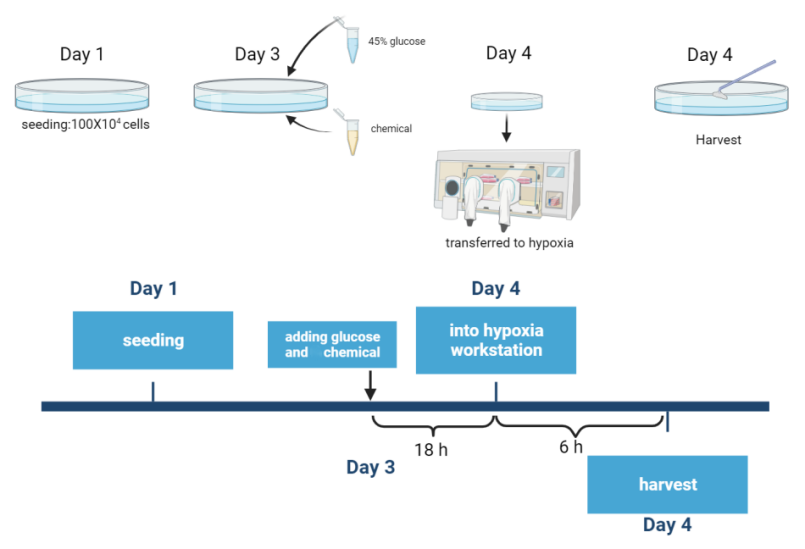


**Supplementary Figure 5** Timeline for nucleo-protein extraction


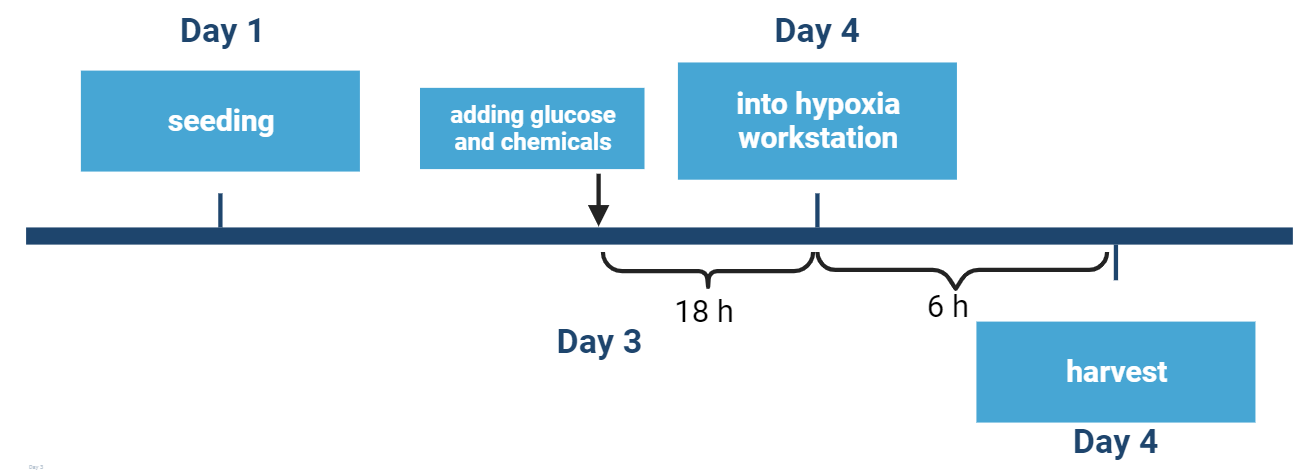


**Supplementary Figure 6** Timeline for immunofluorescence


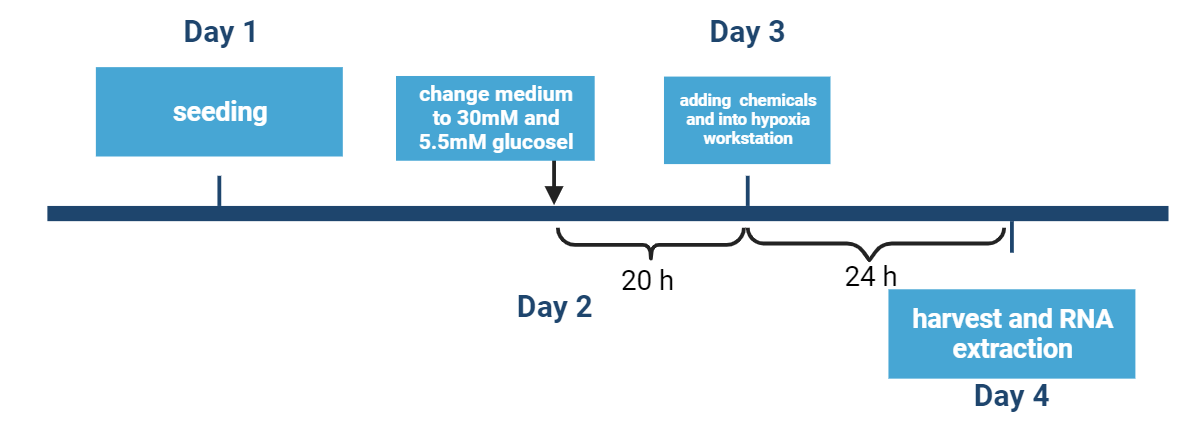


**Supplementary Figure 7** Timeline for qPCR


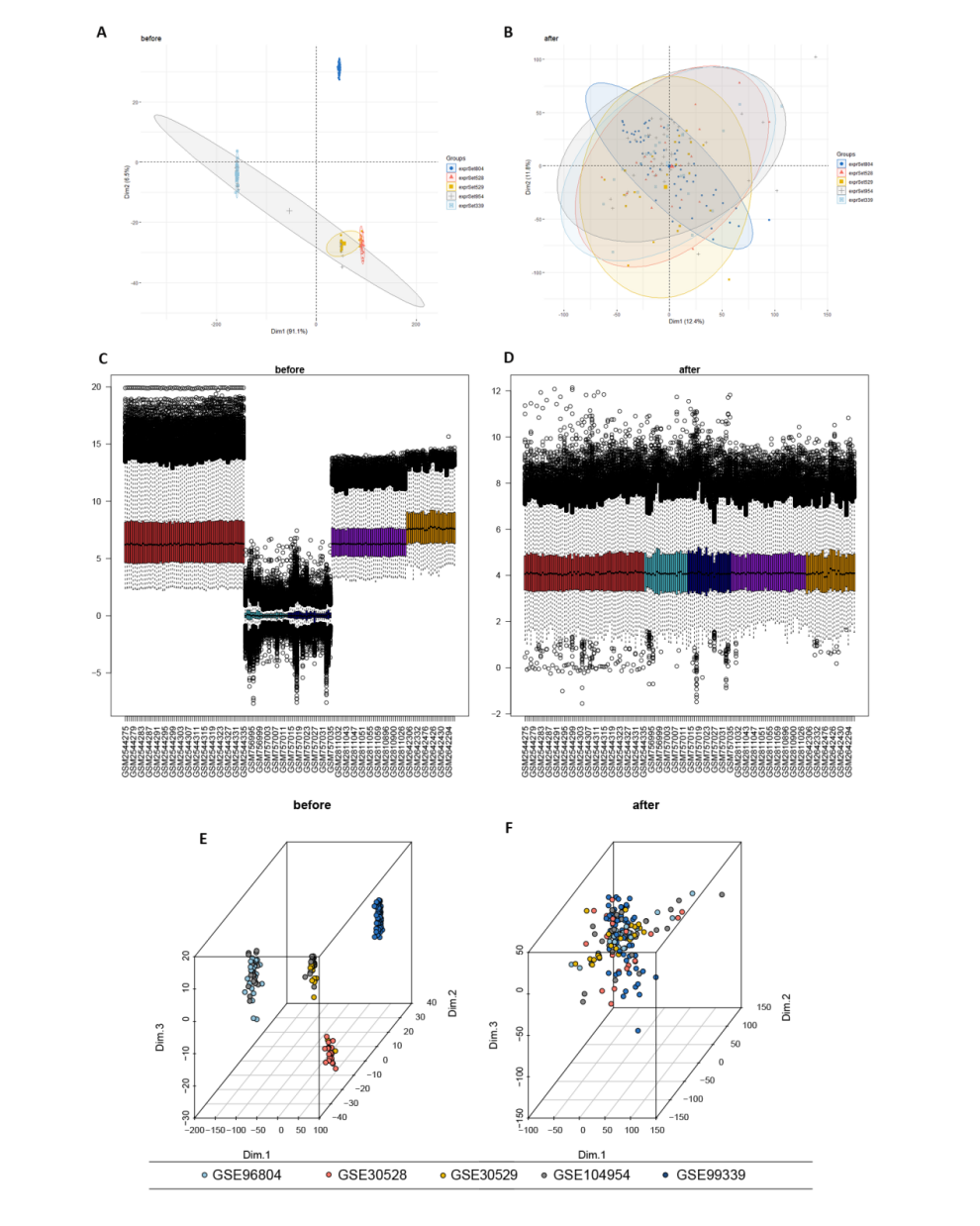


**Supplementary Figure 8**. Data preprocessing. (A, B) PCA of datasets before and after data processing. (C, D) Box plots of datasets before and after data processing. (E, F) 3D projection plots of datasets before and after data processing.


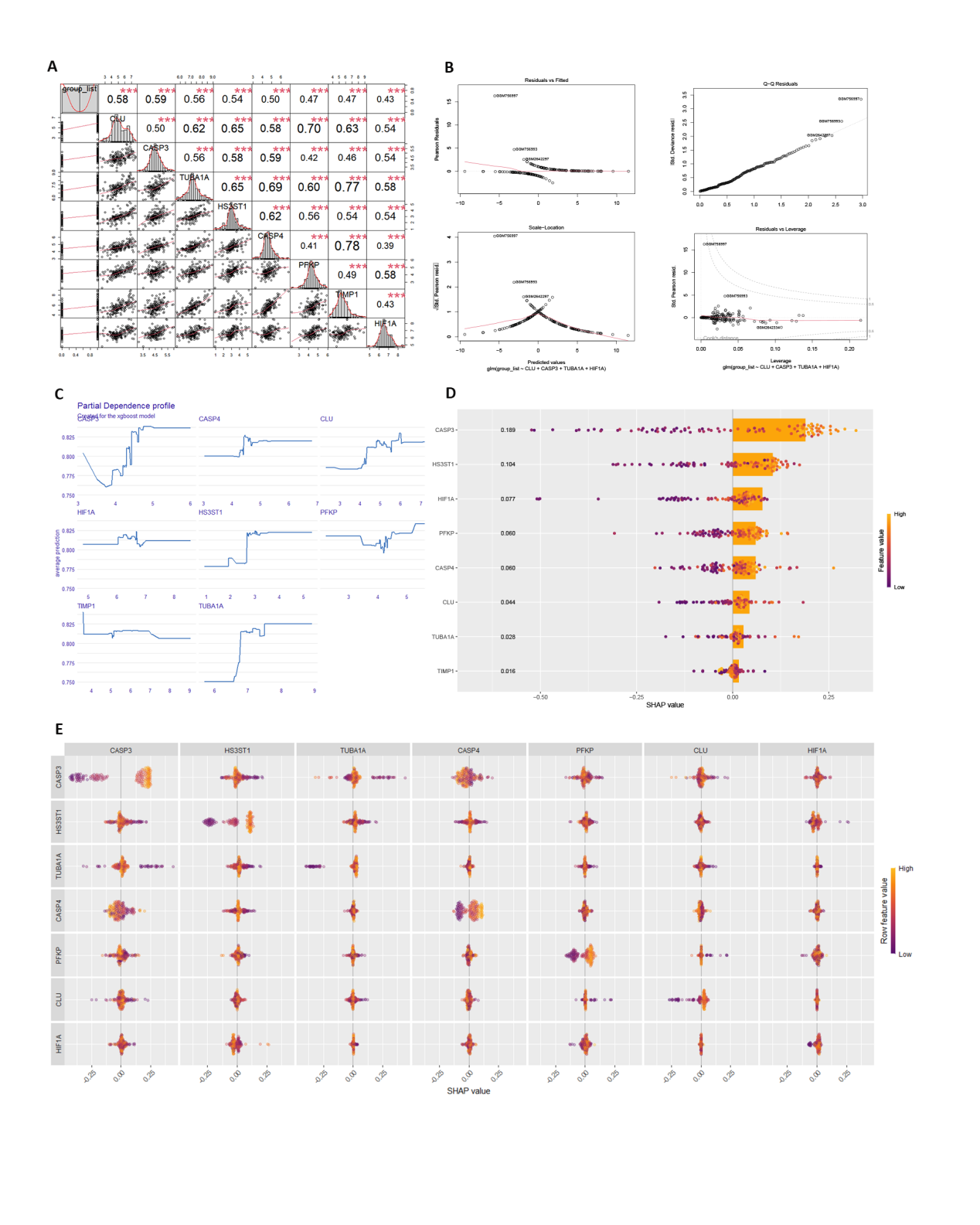


**Supplementary Figure 9**. Stepwise regression and SHAP for XGboost regression. (A, B) Stepwise regression. (C) Partial dependence profile of each variable in XGboost. (D) SHAP for XGboost regression (E) SHAP correlation between 2 variables.


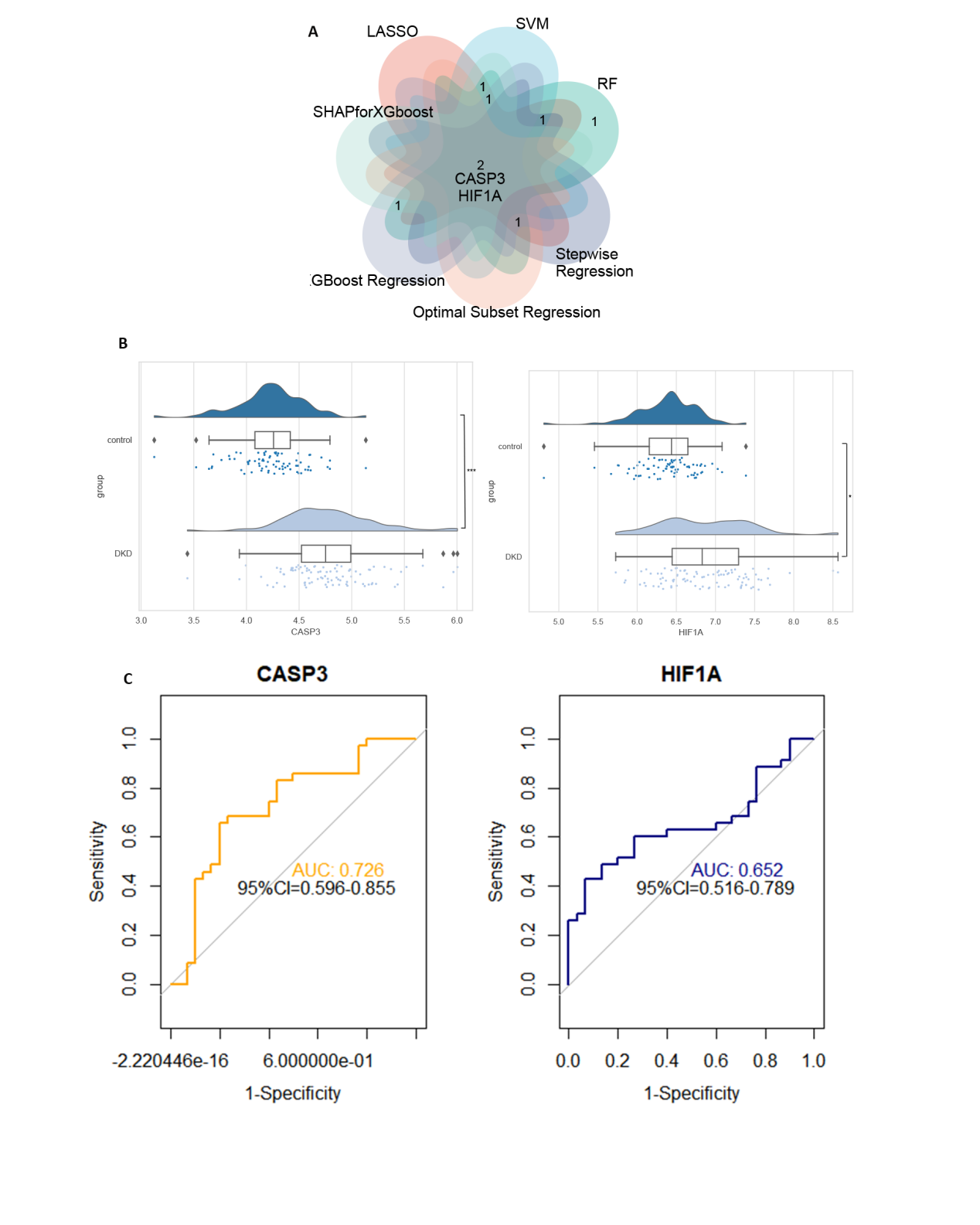


**Supplementary Figure 10.**CASP and HIF-1α as hub genes. (A) Intersection of machine learning results. (B) CASP and HIF-1α expressed significantly in DKD. (C) ROC analysis showed an AUC of 0.726 for CASP3 and 0.652 for HIF-1α in the validation set.


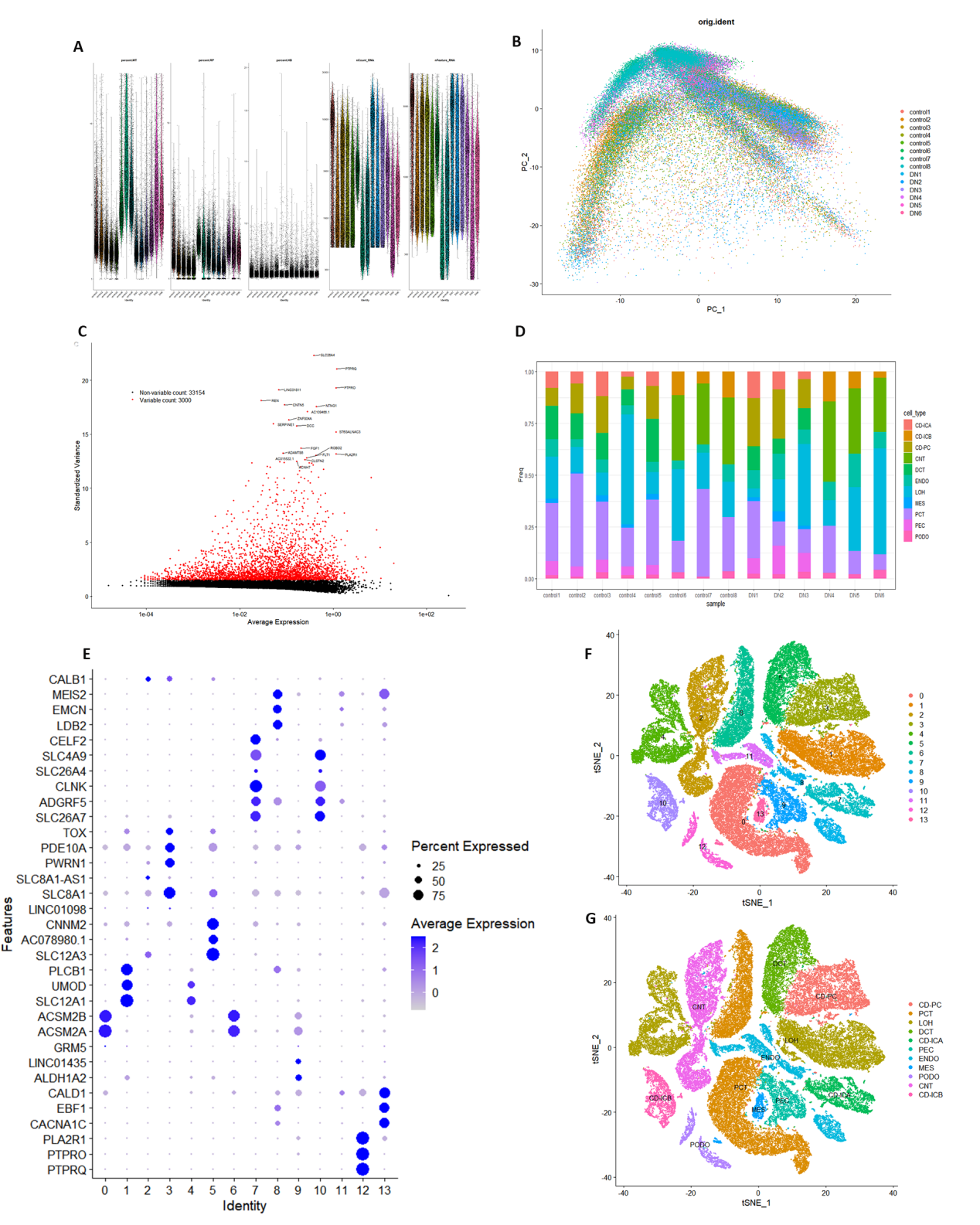


**Supplementary Figure 11.** data control of single-cell transcriptome

**
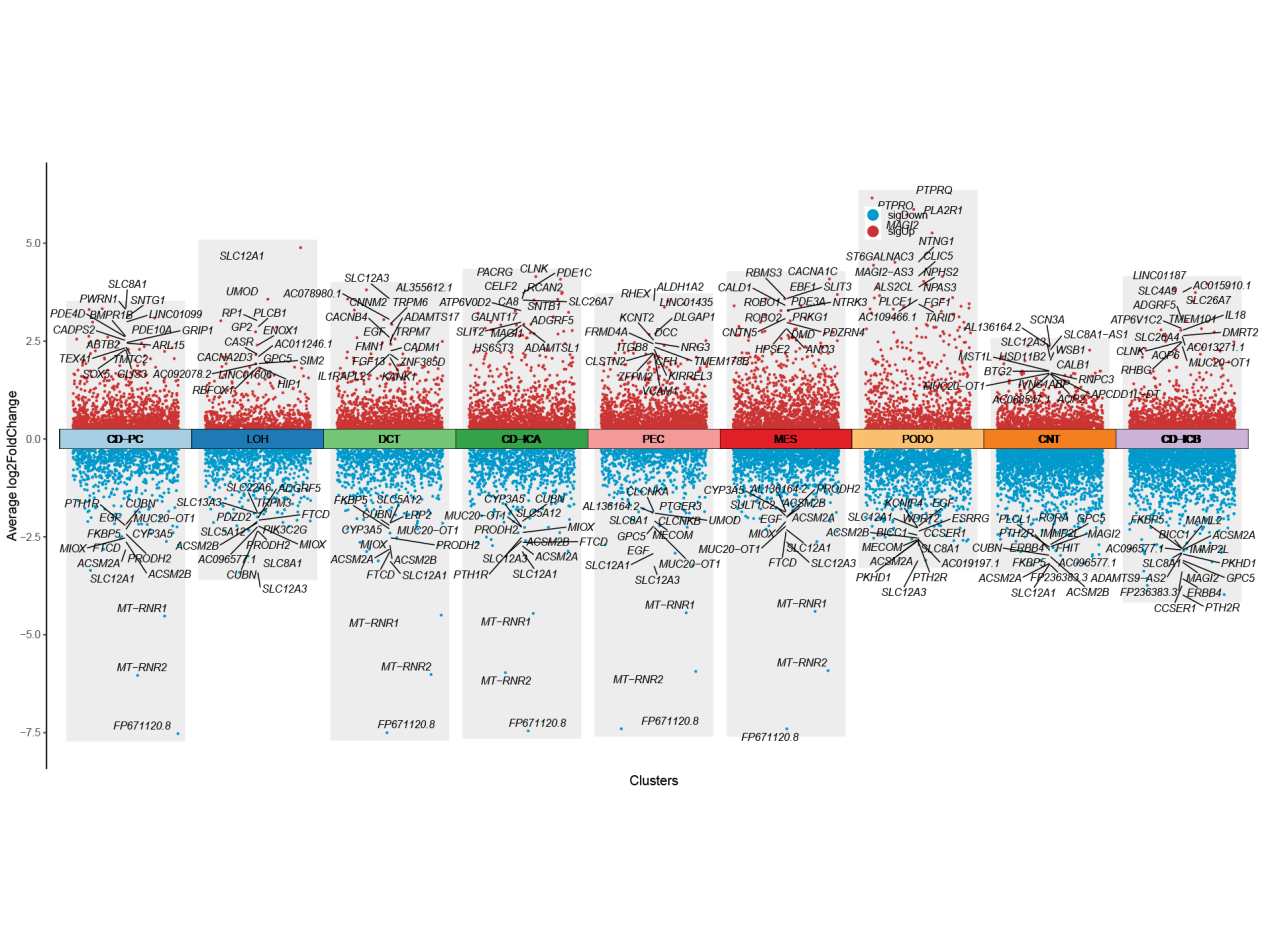
**

**Supplementary Figure 12.** All markers in different cell type


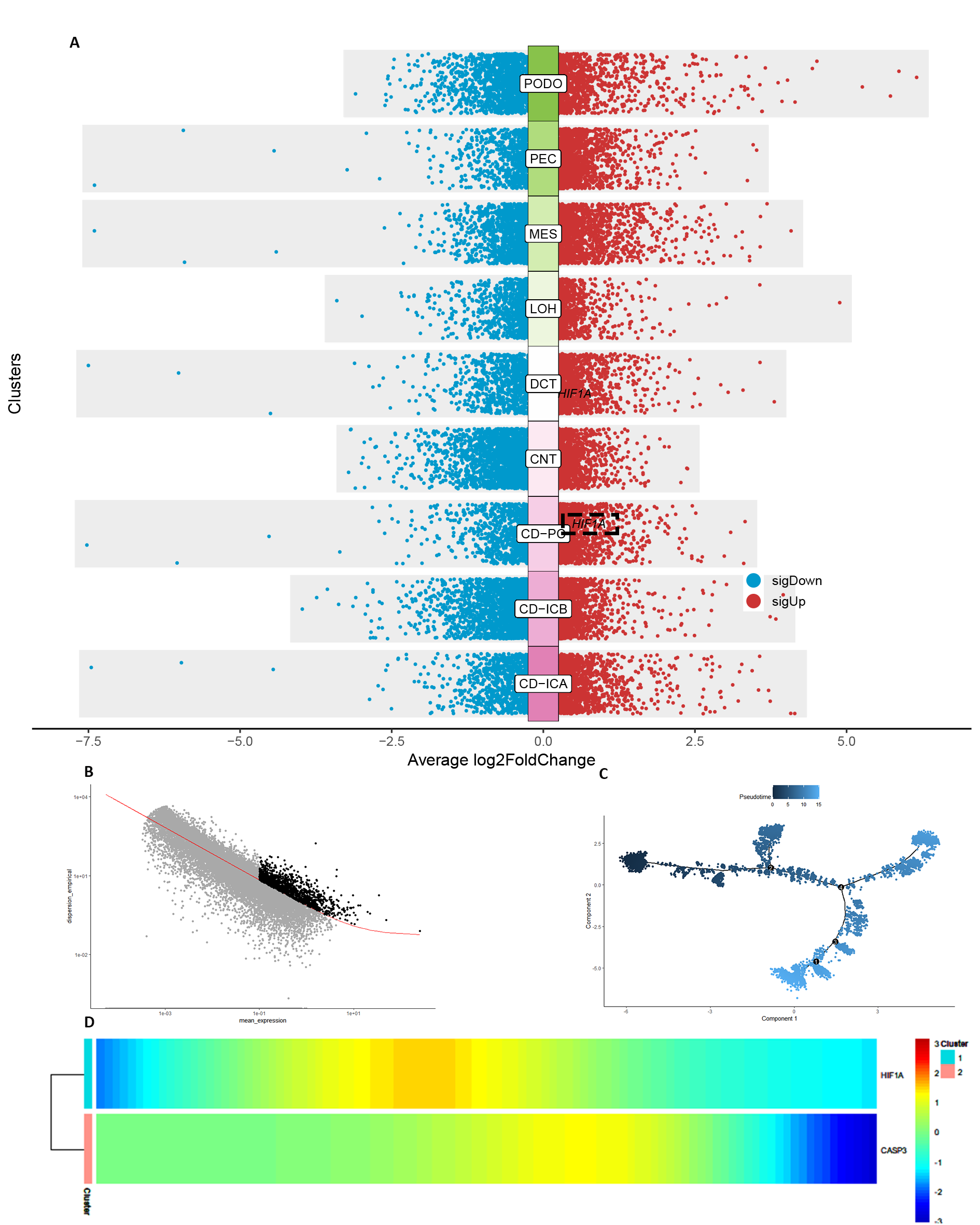


**Supplementary Figure 13.** Differential expression of hub genes and pseudotime analysis of CD-PC Cells. (A) Differential expression of hub genes. (B) Highly variable genes in pseudotime analysis. (C) Differentiation trajectory of CD-PC cells. (D) Expression trajectory of hub genes. Cluster 1: The CD-PC subtype characterized by high HIF1α expression; Cluster 2: The CD-PC subtype characterized by high CASP3 expression.

**
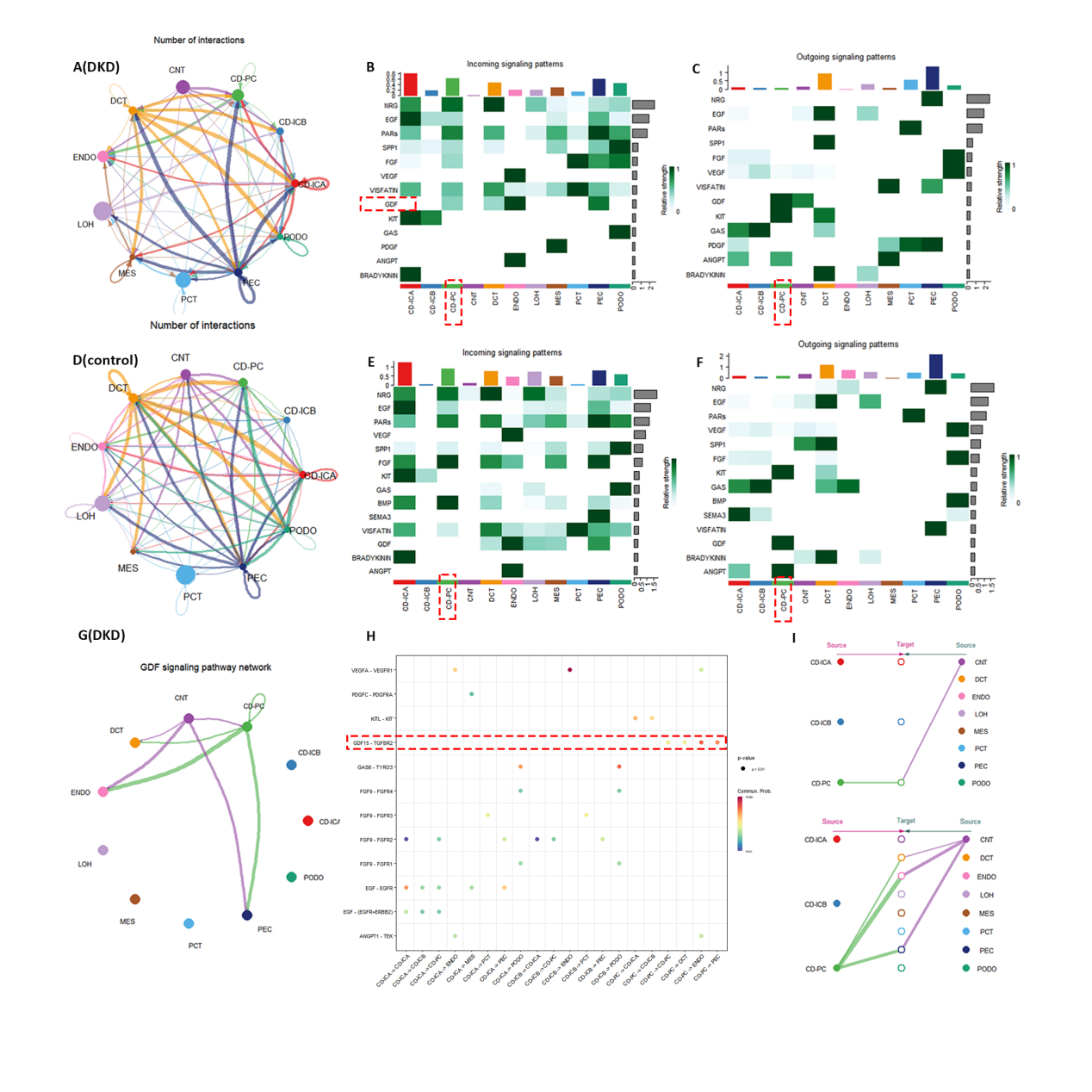
**

**Supplementary Figure 14.** cell communication of CD-PC cells with other cell type. (A, B,C) The total cell communication on DKD samples. (D,E,F) The total cell communication on control samples. (G) GDF signalling pathway in cell communication.


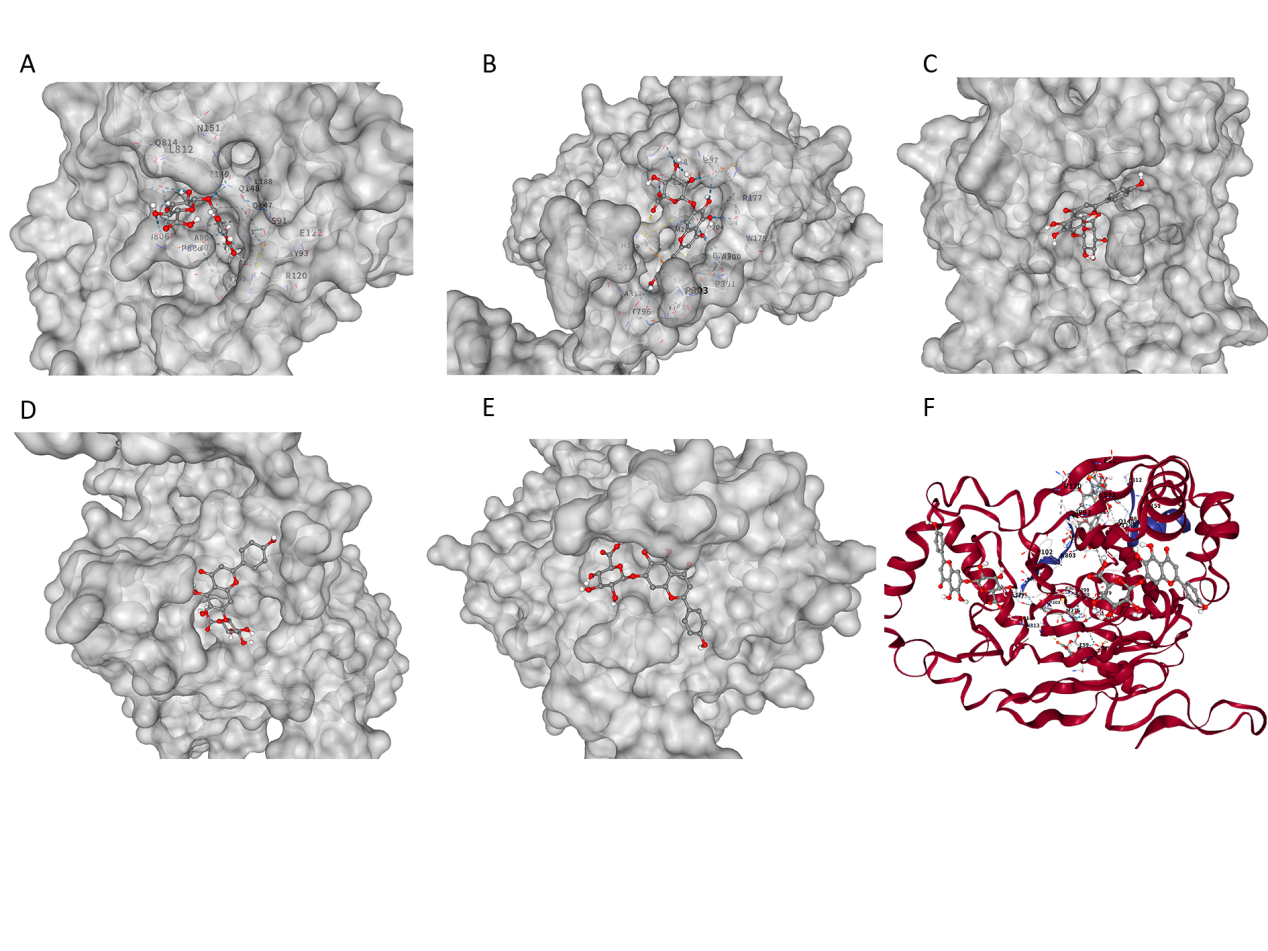


**Supplementary Figure 15** Blind molecular docking based on CB-DOCK2 platform. (A) binding pocket ID-1. (B) binding pocket ID-2. (C) binding pocket ID-3. (D) binding pocket ID-4. (E) binding pocket ID-5. (F) overall binding pocket.


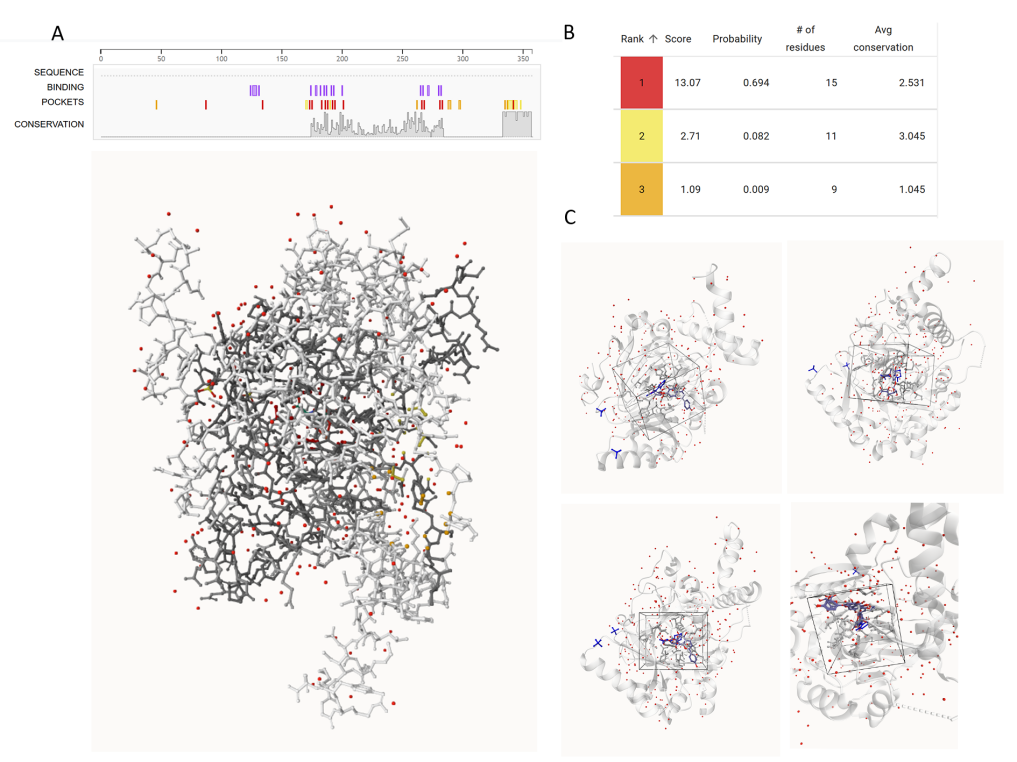


**Supplementary Figure 16** Targeted molecular docking based on P2RANK platform. (A)The natural pocket and binding hot spot formed by the HIF-1α protein. (B) The top three molecular pockets. (C)The embedded scutellarin molecule positions based on the pocket1 structure


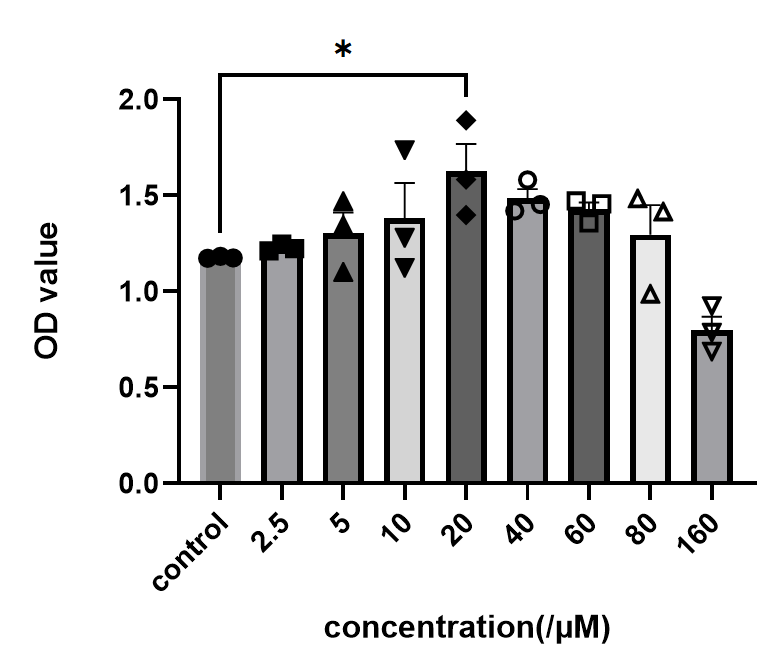


**Supplementary Figure 17** Optimal concentration of scutellarin for mIMCD-3 cell proliferation by MTT assay: 20 μM is the most suitable concentration for cell proliferation. *P (One-way ANOVA) < 0.05


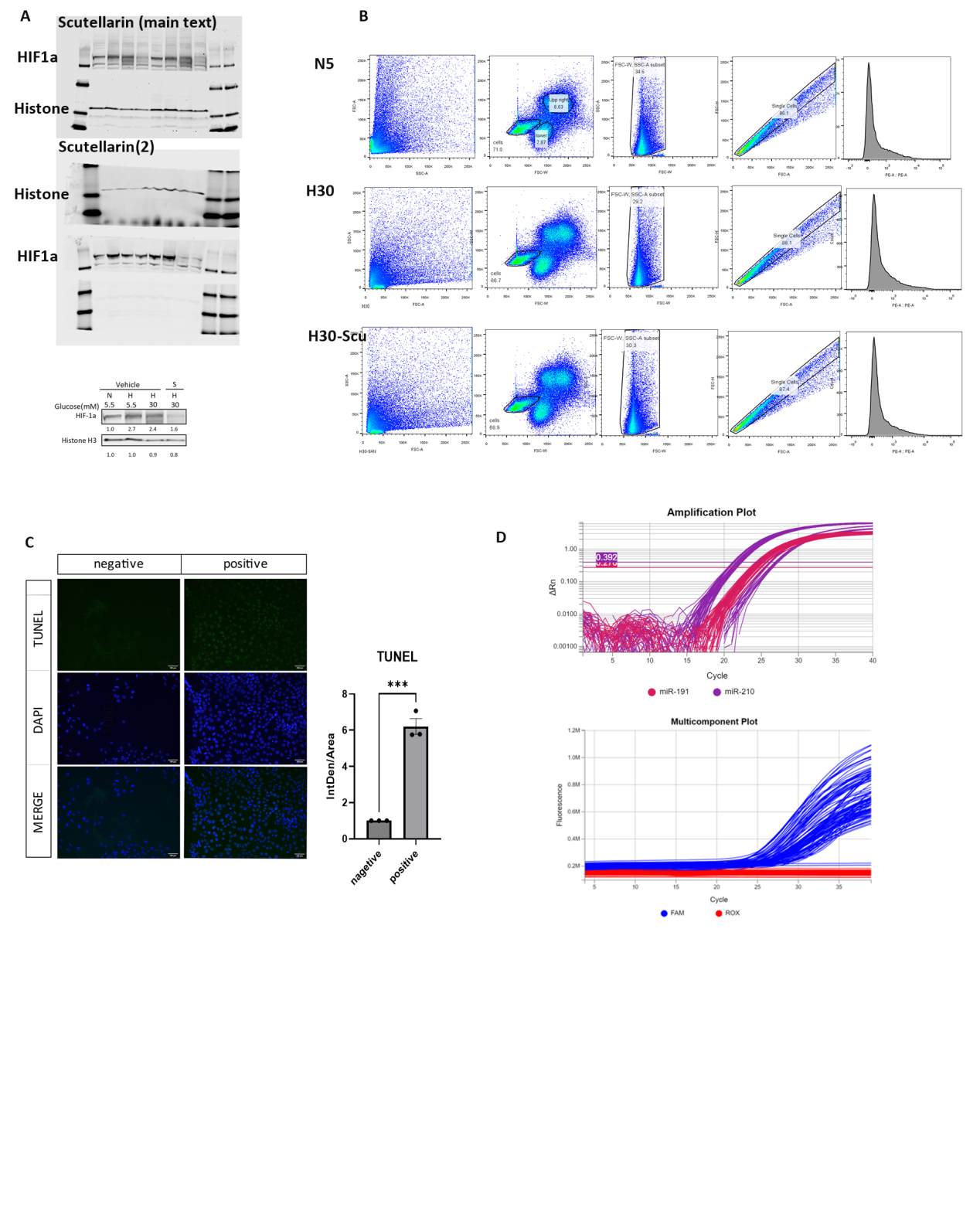


**Supplementary Figure 18. molecular experiment.** (A) WB original blot. (B) Gating strategy of cell cytometry in groups. (C) negative and positive control of TUNEL staning. (D) Amplification plot and multi-component plot of qPCR.

**Supplementary Table 1** Database of network pharmacology

| Database(abbreviation) | Database(http) |
| --- | --- |
| Swisstarget | http://swisstargetprediction.ch/ |
| STITCH | http://stitch.embl.de |
| Uniprot | https://www.uniprot.org/id-mapping |
| DrugBank | https://go.drugbank.com/ |
| TTD | https://db.idrblab.net/ttd/ |
| DAVID | https://david.ncifcrf.gov/ |
| STRING | https://cn.string-db.org/ |
| Metascape | https://metascape.org/ |
| MeSH words | diabetic kidney disease；Nephropathies，Diabetic；Nephropathy，Diabetic；Diabetic Nephropathy；Diabetic Kidney Disease；Diabetic Kidney Diseases；Kidney Disease，Diabetic；Kidney Diseases，Diabetic；Diabetic Glomerulosclerosis；Glomerulosclerosis，Diabetic；Intracapillary Glomerulosclerosis；Nodular Glomerulosclerosis；Glomerulosclerosis，Nodular |

**Supplementary Table 2** Dataset in RNA transcriptome and single-cell transcriptome

| Dataset | Database | Platform | Sample | Tissue |
| --- | --- | --- | --- | --- |
| GSE96804 | GEO-Expression profiling by array | GPL17586 | 41 cases of DKD,  20 cases of control | Glomerulus |
| GSE30528 | GEO-Expression profiling by array | GPL571 | 9 cases of DKD,  13 cases of control | Glomerulus |
| GSE30529 | GEO-Expression profiling by array | GPL571 | 10 cases of DKD,  12 cases of control | Tubules |
| GSE104954 | GEO-Expression profiling by array | GPL24120  GPL22945 | 17 cases of DKD,  21 cases of control | Tubules |
| GSE99339 | GEO-Expression profiling by array | GPL19184  GPL19109 | 14 cases of DKD  11 cases of control | kidney tissue |
| GSE47185 (validation) | GEO-Expression profiling by array | GPL11670  GPL14663 | 32 cases of DKD,  27 cases of control | Glomer and  tubule |
| GSE218344  (validation) | GEO-Expression profiling by array | GPL21827 | 3 cases of DKD,  3 cases of control | kidney tissue |
| GSE131882 | GEO-Single Cell Transcriptomic | GPL24676 | 3 cases of early DKD,  3 cases of controls | kidney tissue |
| GSE195460 | GEO-Single Cell Transcriptomic | [GPL24676](https://www.ncbi.nlm.nih.gov/geo/query/acc.cgi?acc=GPL24676) | 3 cases of early DKD,  5 cases of controls | kidney tissue |

**Supplementary Table 3** Hypoxia and apoptosis geneset

| Origination | Kyoto Encyclopedia of Genes and Genomes (KEGG) database, GSEV-Molecular Signatures Database, Genecard database, DisGeNet database, Reactome database, and Comparative Toxicogenomics Database |
| --- | --- |
| Hypoxia | HIF-1α EGLN1 EGLN3 HIF3A HIF-1αN EGLN2 VEGFA EPAS1 HYOU1 HILPDA VHL ARNT SETD2 HIGD1A EPO HIGD2A CA9 HIGD1B HIGD1C HIGD2B TP53 EP300 HIGD1AP1 CASP3 SLC2A1 P4HTM BNIP3 HIGD1AP18 HIGD1AP13 HIGD1AP16 HIGD1AP11 HIGD1AP5 HIGD1AP10 HIGD1AP12 HIGD1AP15 HIGD1AP2 HIGD1AP3 HIGD1AP4 HIGD1AP8 HIGD1AP14 HIGD1AP17 HIGD1AP6 HIGD1AP7 HIGD1AP9 CREB1 MTOR NHIP EGLN3P1 EGLN1P1 ENSG00000254591 ENSG00000271291 ENSG00000271455 ENSG00000270577 ENSG00000271439 CTNNB1 COL1A1 IGF1 PDK1 CASP9 NOS1 MIR210 TNF IL6 DDIT4 CITED2 FOS EDN1 BCL2 ACKR3 ADM ADORA2B AK4 AKAP12 ALDOA ALDOB ALDOC AMPD3 ANGPTL4 ANKZF1 ANXA2 ATF3 ATP7A B3GALT6 B4GALNT2 BCAN BGN BHLHE40 BNIP3L BRS3 BTG1 CA12 CASP6 CAV1 CAVIN1 CAVIN3 CCN1 CCN2 CCN5 CCNG2 CDKN1A CDKN1B CDKN1C CHST2 CHST3 COL5A1 CP CSRP2 CXCR4 DCN DDIT3 DPYSL4 DTNA DUSP1 EDN2 EFNA1 EFNA3 EGFR ENO1 ENO2 ENO3 ERO1A ERRFI1 ETS1 EXT1 F3 FAM162A FBP1 FOSL2 FOXO3 GAA GALK1 GAPDH GAPDHS GBE1 GCK GCNT2 GLRX GPC1 GPC3 GPC4 GPI GRHPR GYS1 HAS1 HDLBP HEXA HK1 HK2 HMOX1 HOXB9 HS3ST1 HSPA5 IDS IER3 IGFBP1 IGFBP3 ILVBL INHA IRS2 ISG20 JMJD6 JUN KDELR3 KDM3A KIF5A KLF6 KLF7 KLHL24 LALBA LARGE1 LDHA LDHC LOX LXN MAFF MAP3K1 MIF MT1E MT2A MXI1 MYH9 NAGK NCAN NDRG1 NDST1 NDST2 NEDD4L NFIL3 NOCT NR3C1 P4HA1 P4HA2 PAM PCK1 PDGFB PDK3 PFKFB3 PFKL PFKP PGAM2 PGF PGK1 PGM1 PGM2 PHKG1 PIM1 PKLR PKP1 PLAC8 PLAUR PLIN2 PNRC1 PPARGC1A PPFIA4 PPP1R15A PPP1R3C PRDX5 PRKCA PYGM RBPJ RORA RRAGD S100A4 SAP30 SCARB1 SDC2 SDC3 SDC4 SELENBP1 SERPINE1 SIAH2 SLC25A1 SLC2A3 SLC2A5 SLC37A4 SLC6A6 SRPX STBD1 STC1 STC2 SULT2B1 TES TGFB3 TGFBI TGM2 TIPARP TKTL1 TMEM45A TNFAIP3 TPBG TPD52 TPI1 TPST2 UGP2 VLDLR WSB1 XPNPEP1 ZFP36 ZNF292 IL6R STAT3 TLR4 IFNG IFNGR1 IFNGR2 RELA NFKB1 INS EGF INSR IGF1R ERBB2 MAP2K1 MAP2K2 MAPK1 MAPK3 MKNK1 MKNK2 PIK3CA PIK3CD PIK3CB PIK3R1 PIK3R2 PIK3R3 AKT1 AKT2 AKT3 EIF4EBP1 EIF4E EIF4E2 EIF4E1B RPS6KB1 RPS6KB2 RPS6 RBX1 ELOC ELOB CUL2 CREBBP CYBB PLCG1 PLCG2 PRKCB PRKCG CAMK2A CAMK2D CAMK2B CAMK2G TIMP1 LTBR TF TFRC FLT1 ANGPT1 ANGPT2 ANGPT4 TEK NOS2 NOS3 NPPA HK3 HKDC1 PFKM PGK2 LDHAL6A LDHAL6B LDHB PDHA2 PDHA1 PDHB |
| Apoptosis | TNFSF10 TNFRSF10A TNFRSF10B FASLG FAS FADD TNF TNFRSF1A TRADD CFLAR CASP8 CASP10 CASP6 CASP3 CASP7 BID BAX BAK1 DIABLO SEPTIN4 HTRA2 CYCS APAF1 CASP9 PRF1 GZMB TUBA1B TUBA4A TUBA3C TUBA1A TUBA1C TUBA8 TUBA3E TUBA3D TUBAL3 MCL1 ACTG1 ACTB SPTA1 SPTAN1 LM LMNB1 LMNB2 PARP1 PARP2 PARP3 PARP4 DFFA DFFB ENDOG AIFM1 ERN1 TRAF2 ITPR1 ITPR2 ITPR3 CAPN1 CAPN2 CASP12 EIF2AK3 EIF2S1 ATF4 DDIT3 CTSB CTSC CTSD CTSF CTSH CTSK CTSL CTSO CTSS CTSV CTSW CTSZ BIRC2 BIRC3 XIAP BIRC5 BCL2L11 BCL2L1 BCL2 DAXX RIPK1 DAB2IP MAP3K5 MAPK8 MAPK10 MAPK9 BAD JUN FOS TP53 HRK MAP3K14 CHUK IKBKB IKBKG NFKBIA NFKB1 RELA PTPN13 GADD45A GADD45B GADD45G TRAF1 BCL2A1 ATM PIDD1 TP53AIP1 BBC3 PMAIP1 CASP2 NGF NTRK1 IL3 IL3RA CSF2RB PIK3CA PIK3CD PIK3CB PIK3R1 PIK3R2 PIK3R3 PDPK1 AKT1 AKT2 AKT3 HRAS KRAS NRAS RAF1 MAP2K1 MAP2K2 MAPK1 MAPK3 ADD1 AIFM3 ANKH ANXA1 APP ATF3 AVPR1A BCAP31 BCL10 BCL2L10 BCL2L2 BGN BIK BMF BMP2 BNIP3L BRCA1 BTG2 BTG3 CASP1 CASP4 CAV1 CCNA1 CCND1 CCND2 CD14 CD2 CD38 CD44 CD69 CDC25B CDK2 CDKN1A CDKN1B CLU CREBBP CTH CTNNB1 CYLD DAP DAP3 DCN DNAJA1 DNAJC3 DNM1L DPYD EBP EGR3 EMP1 ENO2 ERBB2 ERBB3 EREG ETF1 F2 F2R FDXR FEZ1 GCH1 GNA15 GPX1 GPX3 GPX4 GSN GSR GSTM1 GUCY2D H1-0 HGF HMGB2 HMOX1 HSPB1 IER3 IFITM3 IFNB1 IFNGR1 IGF2R IGFBP6 IL18 IL1A IL1B IL6 IRF1 ISG20 KRT18 LEF1 LGALS3 LMNA LUM MADD MGMT MMP2 NEDD9 NEFH PAK1 PDCD4 PDGFRB PEA15 PLAT PLCB2 PLPPR4 PPP2R5B PPP3R1 PPT1 PSEN1 PSEN2 PTK2 RARA RETSAT RHOB RHOT2 RNASEL ROCK1 SAT1 SATB1 SC5D SLC20A1 SMAD7 SOD1 SOD2 SQSTM1 TAP1 TGFB2 TGFBR3 TIMP1 TIMP2 TIMP3 TNFRSF12A TOP2A TSPO TXNIP VDAC2 WEE1 |

**Supplementary Table 4** materials and equipment

| Reagent type  (species) or  resource | Designation | Source or  reference | Identifiers | Additional information |
| --- | --- | --- | --- | --- |
| MTT |  |  |  |  |
| Commercial assay  or kit | Vybrant® MTT Cell Proliferation Assay Kit | Molecular Probes | Cat#: V-13154 |  |
|  | 96-well plates | Thermofisher | Cat#: 92697 |  |
|  | phosphate buffered saline（PBS） | Gibco | Cat#: 10010002 | Dilute 10X to 1X with autoclaved MQ-H2O |
| Medium | Roswell Park Memorial Institute （RPMI） 1640 medium | Gibco | Cat#: 32404014 |  |
| Medium | Dulbecco’s Modified Eagle’s  Medium | Thermo Fisher  Scientific | Cat#: 31885-023 |  |
| Equipment | Multiskan SkyHigh Microplate Spectrophotometer | Thermo Fisher  Scientific | Cat#: A51119700DPC |  |
| TUNEL |  |  |  |  |
| Commercial assay or kit | In Situ Cell Death  Detection Kit, POD | Roche | Cat. No:  11684817910 |  |
| Chemical | Triton X-100 | Sigma-Aldrich | Cat.No:[9036-19-5](https://www.sigmaaldrich.com/SE/en/search/9036-19-5?focus=products&page=1&perpage=30&sort=relevance&term=9036-19-5&type=cas_number) |  |
| Chemical | wide-mouth bottles, PFA | BRAND | Cat.No:BR128754-1EA |  |
| Chemical | 3% H2O2 in methanol | Sigma-Aldrich | Cat.No:925B-03 |  |
| Chemical | DNase I, RNase-free （1 U/µL） | ThermoFisher | Cat. No. EN0521 | Not frozen repeatedly |
| Chemical | Prolong Gold Antifade Mountant with DAPI | Invitrogen | Cat. No:P36931 |  |
| Equipment | Leica dm3000 led Microscope with shutter | Leica | Cat. No. 10052-524 | Channel 1-5 |
| Caspase 3/7 assay |  |  |  |  |
| Commercial assay or kit | Caspase-Glo  3/7 assay （10 mL） | Promega | Cat. No:G8091 | protected from light |
|  | 96-well plates | Thermofisher | Cat#: 92697 |  |
| equipment | GloMax | Promega |  | With caspase 3/7 system |
| equipment | Orbital shakers | ThermoFisher | Cat. No:SHKE416HP |  |
| EPR |  |  |  |  |
| buffer | Krebs-Henseleit buffer | Sigma-Aldrich | Cat:#K3753 |  |
| Spin probe | mito-TEMPO-H | Enzolifesciences | Cat:#ALX-430-171-M005 |  |
| Chemical | antimycin A | Thermo Scientific Chemicals | Cat:#J63522.MA |  |
| equipment | Benchtop EPR with the Bruker e-scan | Bruker | Cat:#T157941 | With Xepr and WinEprSuite Software |
| MitoSox cytometry |  |  |  |  |
| buffer | MACS BSA Stock Solution | Miltenyibiotec | cat:#130-091-376 |  |
| buffer | autoMACS Rinsing Solution | Miltenyibiotec | cat:#130-091-222 |  |
| Commercial assay  or kit | MitoSOX Red Mitochondrial  Superoxide Indicator, for live cell imaging | ThermoFisher | cat: M36008 | 396/610 nm |
| Equipment | CyAn™ ADP Analyzer by Beckman Coulter Life Sciences | Beckman Coulter | cat:#0000050K |  |
| Luciferase reporter system |  |  |  |  |
| Cell line （Mus  musculus） | mIMCD-3 cell line | ATCC | Cat#:  CRL-2123;  RRID:CVCL_0429 |  |
| Commercial assay or kit | MycoAlert PLUS mycoplasma  detection kit | LONZA | Cat#:  LT07-218 |  |
| Commercial assay  or kit | Dual-Luciferase Reporter  Assay System | Promega | Cat#: E1910 |  |
| Commercial assay | Lipofectamine™ 3000 Transfection Reagent | Thermo Fisher  Scientific | Cat#: L3000001 |  |
| Commercial assay  or kit | ZymoPURE II Plasmid Maxiprep Kit | Nordic Biosite | Cat#: BioSite-D4202 |  |
| competent cells | NEB® Stable Competent E. coli （High Efficiency） | New England BioLabs | Cat#: C3040H |  |
| powder | LB Broth with Agar（Miller） | Sigma | Cat#: L3147 |  |
| Medium | Dulbecco’s Modified Eagle’s  Medium | Thermo Fisher  Scientific | Cat#: 31885-023 |  |
| Medium | Fetal Bovine Serum, qualified, heat inactivated | Gibco | Cat#: 16140071 |  |
| other | Trypsin-EDTA （0.05%）, phenol red | Gibco | Cat#: 25300104 | Thawed when using |
| other | phosphate buffered saline | Gibco | Cat#: 10010002 |  |
| other | 45% D-（+）-Glucose solution | Sigma aldrich | Cat#: G8769 |  |
| Chemical | Scutellarin | Divbio Science | Cat#: S3810 | dissolved in DMSO |
| Chemical | Dimethyloxalylglycine  （DMOG） | Divbio Science | Cat#: S7483 |  |
| other | 12-well plate | Sigma | Cat#:Z707783 |  |
| other | Hypoxia Workstation  INVIVO2 （Ruskinn）. | LAF Technologies Pty Ltd |  |  |
| other | Round-Bottom Polypropylene Test Tubes With Cap | Falcon™ | Cat#:352059 |  |
| Western-blot |  |  |  |  |
| Antibody | anti-HIF-1alpha （Rabbit  polyclonal） | Novus  Biologicals | Cat#: NB100-479;  RRID:AB_10000633 | WB: 1:500 |
| Antibody | anti-Histone H3 （Rabbit  polyclonal） | Abcam | Cat#: ab1791;  RRID:AB_302613 | WB: 1:5,000 |
| Antibody | IRDye 800 goat anti-rabbit  Secondary Antibody | LI_COR  Biosciences | Cat#:925-32211;  RRID:AB_2651127 | WB:1:20,000 |
| Other | NuPAGE™ LDS Sample Buffer （4X） | Invitrogen™ | Cat#:NP0007 |  |
| Chemical | DTT （dithiothreitol） | Thermo Scientific | Cat#:R0861 |  |
|  | Halt™ Protease and Phosphatase Inhibitor Cocktail （100X） | Thermo Scientific | Cat#:78440 |  |
|  | Bradford | Thermo Scientific | Cat#:23236 |  |
|  | BSA standard ampule | Thermo Scientific | Cat#:23209 |  |
|  | MOPS running buffer | Novex | Cat#:NP0001 |  |
|  | Surepage gel | GenScript | Cat#:M00656 |  |
| Marker | Seeblue | invitrogen | Cat#:LC5925 |  |
| buffer | NuPage Antioxidant | Thermo Fisher, | Cat#:NP0005 |  |
|  | Immun-Blot® Low Fluorescence PVDF | BioRAD | Cat#:162-0260 |  |
|  | 20 X transfer buffer | Novex | Cat#:NP0006-1 |  |
|  | filter paper | Thermo scientific | Cat#:88600 |  |
|  | Intercept blocking buffer（TBS） | Li-Cor | Cat#:927-60001 |  |
| tool | Glass Dounce homogenizer | [Bellco Glass](https://se.vwr.com/store/supplier/id/BELC/bellco-glass) | Cat#:BELC1984-10007 |  |
|  | Slide-A-Lyzer™ MINI Dialysis Device, 7K MWCO | Thermo Scientific | Cat#:69560 |  |
| Immunofluorescence |  |  |  |  |
| buffer | 4% Formaldehyde | Sigma | Cat#: [F8775](https://www.sigmaaldrich.com/SE/en/product/sigma/f8775) |  |
| antibody | Rabbit polyclonal anti-HIF1α antibody | GeneTex | Cat#:GTX127309 | recycled |
| antibody | Goat anti-Rabbit Alexa 594 | ThermoFisher Scientific | Cat#:A-11037 |  |
|  | ProLong Gold Antifade Mountant with DAPI | Invitrogen | Cat. No:P36931 |  |
| qPCR |  |  |  |  |
| Commercial kit | NucleoSpin miRNA kit | Techtum （Macherey-Nagel） | Cat#: 740971.50 |  |
|  | Taqman Advanced  miRNA Assays477952_mir/hsa-miR-191 | ThermoFisher | Cat#: A25576 | CAACGGAAUCCCAAAAGCAGCUG |
|  | Taqman Advanced  miRNA Assaays477970_mir/hsa-miR-210-3p | ThermoFisher | Cat#: A25577 | CUGUGCGUGUGACAGCGGCUGA |
|  | TaqMan Fast Advanced MasterMix | ThermoFisher | Cat#: 4444963 |  |
| Commercial kit | TaqMan Advanced miRNA  cDNA Synthesis Kit | ThermoFisher | Cat#: A28007 |  |
|  | High capacity cDNA  reverse transcriptase kit | ThermoFisherSci | Cat#:4368814 |  |
|  | MicroAmp™ Optical  384-Well Reaction Plate with Barcode | ThermoFisher | Cat#:4309849 |  |

**Supplementary Table 5** Molecular docking energy based on Autodock

| **Chemical** | **MOLID** | **conformation locus** | **binding energy** | **ligand**  **efficiency** | **Intermol**  **energy** | **Electrostatic**  **Energy** | **RefRMS** | **Hydrogen bonds** | **torsional energy** |
| --- | --- | --- | --- | --- | --- | --- | --- | --- | --- |
| scutellarin | MOL002931 | 1 | -5.42 | -0.27 | -4.31 | -0.15 | 13.08 | 1(GLN663) | 0.89 |
|  |  | 2 | -5.22 | -0.21 | -3.12 | -0.05 | 11.03 | 1(LYS674) |  |
|  |  | 3 | -5.17 | -0.21 | -3.06 | -0.01 | 17.31 | 0 |  |
|  |  | 4 | -5.06 | -0.20 | -2.96 | -0.11 | 17.60 | 0 |  |
|  |  | 5 | -5.04 | -0.20 | -2.93 | -0.03 | 15.61 | 0 |  |
|  |  | 6 | -4.90 | -0.20 | -2.80 | -0.03 | 13.66 | 0 |  |
|  |  | 7 | -4.88 | -0.19 | -2.77 | -0.02 | 22.64 | 1(LEU783) |  |
|  |  | 8 | -3.87 | -0.19 | -2.77 | -0.14 | 14.33 | 0 |  |
|  |  | 9 | -2.84 | -0.19 | -2.73 | -0.13 | 12.50 | 0 |  |
|  |  | 10 | -2.82 | -0.19 | -2.72 | -0.06 | 14.95 | 0 |  |

**Supplementary Table 6** Molecular docking energy based on CB-DOCK2

| **ID** | **Cavities_volume** | **center_x** | **center_y** | **center_z** | **size_x** | **size_y** | **size_z** | **score** | **contact_residues** |
| --- | --- | --- | --- | --- | --- | --- | --- | --- | --- |
| 1 | 1464 | 27.578 | 21.209 | 37.363 | 24 | 24 | 24 | -7.7 | ASP:89:A,PHE:90:A,SER:91:A,TYR:93:A,TYR:102: |
| 2 | 680 | 36.614 | 31.932 | 20.139 | 24 | 24 | 24 | -8.3 | ILE:56:A,GLU:57:A,ASN:58:A,GLU:59:A,GLU:60:A |
| 3 | 433 | 2.007 | 22.356 | 34.366 | 24 | 24 | 24 | -6.9 | LEU:78:A,GLU:79:A,VAL:129:A,GLU:130:A,LYS:131:A |
| 4 | 167 | 15.799 | 35.506 | 37.523 | 24 | 24 | 24 | -7.7 | THR:97:A,LYS:99:A,PHE:100:A,LEU:101:A,PHE:111:A |
| 5 | 162 | 20.034 | 25.624 | 30.812 | 24 | 24 | 24 | -6.5 | SER:91:A,TYR:93:A,TYR:102:A,ARG:120:A,GLU:122:A |

**Supplementary Table 7** Molecular docking energy based on P2RANK

| **name** | **rank** | **score** | **probability** | **sas_points** | **surf_atoms** | **center_x** | **center_y** | **center_z** | **residue_ids** |
| --- | --- | --- | --- | --- | --- | --- | --- | --- | --- |
| pocket1 | 1 | -13.07 | 0.694 | 26 | 24 | 20.4882 | 25.2594 | 29.5789 | A_100 A_147 A_186 A_188 A_196 A_199 A_201 A_205 A_207 A_214 A_279 A_281 A_294 A_296 S_803 |
| pocket2 | 2 | -2.71 | 0.082 | 36 | 19 | 30.8388 | 20.8879 | 33.8187 | A_183 A_184 A_203 S_799 S_800 S_801 S_802 S_804 S_805 S_806 S_814 |
| pocket3 | 3 | -1.09 | 0.009 | 17 | 15 | 39.238 | 31.7911 | 23.8861 | A_275 A_301 A_302 A_303 A_313 A_314 A_59 S_796 S_798 |
